# Integration of Biochemical, Biophysical and Transcriptomics Data for Investigating the Structural and Nanomechanical Properties of the Yeast Cell Wall

**DOI:** 10.3389/fmicb.2017.01806

**Published:** 2017-09-27

**Authors:** Marion Schiavone, Sébastien Déjean, Nathalie Sieczkowski, Mathieu Castex, Etienne Dague, Jean M. François

**Affiliations:** ^1^Laboratoire d'Ingénierie des Systèmes Biologiques et Procédés, Institut National des Sciences Appliquées de Toulouse, UPS, INP, Université de Toulouse Toulouse, France; ^2^Lallemand SAS Blagnac, France; ^3^Institut de Mathématiques de Toulouse Toulouse, France; ^4^Laboratoire D'analyse et D'architecture des Systèmes du-Centre National de la Recherche Scientifique, Université de Toulouse Toulouse, France

**Keywords:** cell wall, β-glucans, mannans, chitin, yeast, atomic force microscopy, microarrays, multivariate analysis

## Abstract

The yeast cell is surrounded by a cell wall conferring protection and resistance to environmental conditions that can be harmful. Identify the molecular cues (genes) which shape the biochemical composition and the nanomechanical properties of the cell wall and the links between these two parameters represent a major issue in the understanding of the biogenesis and the molecular assembly of this essential cellular structure, which may have consequences in diverse biotechnological applications. We addressed this question in two ways. Firstly, we compared the biochemical and biophysical properties using atomic force microscopy (AFM) methods of 4 industrial strains with the laboratory sequenced strain BY4743 and used transcriptome data of these strains to infer biological hypothesis about differences of these properties between strains. This comparative approach showed a 4–6-fold higher hydrophobicity of industrial strains that was correlated to higher expression of genes encoding adhesin and adhesin-like proteins and not to their higher mannans content. The second approach was to employ a multivariate statistical analysis to identify highly correlated variables among biochemical, biophysical and genes expression data. Accordingly, we found a tight association between hydrophobicity and adhesion events that positively correlated with a set of 22 genes in which the main enriched GO function was the sterol metabolic process. We also identified a strong association of β-1,3-glucans with contour length that corresponds to the extension of mannans chains upon pulling the mannosyl units with the lectin-coated AFM tips. This association was positively correlated with a group of 27 genes in which the seripauperin multigene family was highly documented and negatively connected with a set of 23 genes whose main GO biological process was sulfur assimilation/cysteine biosynthetic process. On the other hand, the elasticity modulus was found weakly associated with levels of β-1,6-glucans, and this biophysical variable was positively correlated with a set of genes implicated in microtubules polymerization, tubulin folding and mitotic organization.

## Introduction

The yeast *Saccharomyces cerevisiae* is surrounded by a 100–150 nm thick armor termed the cell wall which amounts to 10–25% of the cell dry mass (Aguilar-Uscanga and Francois, 2003; Yin et al., 2007). In transmission electron microscopy, the cell wall appears as a two layered structure. The fuzzy electron dense outer layer of about 30–40 nm thick is supposed to mainly contain cell wall mannoproteins, whereas the internal layer of about 70–100 nm that shields the plasma membrane is mainly made of β-glucans (Osumi, 1998; Backhaus et al., 2010; Schiavone et al., 2016). In spite of these apparent distinct layers, the four macromolecules that composed the cell wall, namely mannoproteins, β-1, 3 glucans, β-1,6 glucans and chitin are covalently joined to generate a highly dynamic three-dimensional architecture of the wall. In particular, β-1, 6-glucans have a role in cross-linking cell wall protein (CWPs) to β-1,3-glucans via the connection with the remnant glycosylphosphatidyl inositol (GPI) anchor attached to these types of proteins. Another type of cell wall protein termed PIR-CWPs are directly linked to β-1,3-glucans through γ-carboxylic group of glutamates, whereas the linkage between non reducing end of β-1,3-glucans to the non-reducing end of chitin is determinant for the bud neck formation (Cabib et al., 2012). Besides, chitin is bound to β-1,6 glucans, which in turn is linked to β-1,3-glucans of the lateral walls (Klis et al., 2006; Orlean, 2012). The dynamic nature of the cell wall is witnessed by the ability of yeast cell to show important morphogenetic modifications (as for instance shmoos formation during mating) and to adapt to environmental stress or various injuries caused by drugs, lytic enzymes as well as mutations that cause defect in genes implicated in its synthesis. Genetic analyses led to the finding that the MAPkinase cascade dependent on PKC1 is the main, but not the sole signaling pathway that controls the cell wall dynamic (Lesage and Bussey, 2006; Levin, 2011). In addition, genome wide transcriptomic analysis of this cell wall remodeling caused by mutations in genes specifically implicated in synthesis of cell wall or by cell wall perturbing agents led to the identification of a core of about 50 upregulated genes that are considered to be critically important in this cellular response (Lagorce et al., 2003; Garcia et al., 2004). Among them were identified several genes encoding glycosyltransferase/hydrolase, that were called “cell wall remodeling enzymes.” These changes at the transcriptional level can be associated with biochemical modifications that take place in response to cell wall remodeling and which are: (i) an increase of chitin amount in cell wall which can contribute up to 20% of the cell wall when important genes encoding for its biosynthesis are deleted; (ii) a modification of linkages between cell wall components and (iii) an increase of cell wall remodeling enzymes accompanied with a redistribution of cell wall synthesis and repair machinery (Klis et al., 2006; Orlean, 2012).

The emergence of atomic force microscopy (AFM), initially invented by Binnig, Gerber and Quate at IBM Zurich (Binnig and Quate, 1986) has readily evolved to turn out to be a suitable and versatile tool for probing the physical properties of microbial cell surfaces in their natural liquid environment. Besides imaging the topology of living cells (Ahimou et al., 2002; Dague et al., 2007), nanomechanical properties of the yeast cell wall such as elasticity modulus (or Young's modulus) can be determined from indentation measurement (Alsteens et al., 2008; Arfsten et al., 2010). In addition, the single molecule force spectroscopy (SMFS), technique that uses a chemically modified AFM-tip allows to directly monitor specific interaction at the cell surface. As a relevant example is the presence of adhesive patches forming nanodomains at the yeast cell surface as identified by AFM-tip functionalized with concanavalin (Alsteens et al., 2010; Schiavone et al., 2015) Likewise, mapping of cell wall proteins at the cell surface can be monitored using corresponding antibodies covalently fixed on the AFM-tips (Formosa et al., 2015a).

Each of the polysaccharides that constitute the yeast cell wall exhibits relevant technological properties. The cell wall mannoproteins are responsible of the adhesion properties of yeast to inert surface, the formation of biofilms (Blankenship and Mitchell, 2006; Bojsen et al., 2012) and have been reported to retain aroma and phenolic compounds (Chalier et al., 2007; Pradelles et al., 2008). Also, yeast mannoproteins have been extensively studied in the field of animal and fish nutrition for their so called prebiotic properties and their ability to bind to potential pathogenic bacteria through direct linkage with mannose specific fimbriae present at the bacteria surface, hence limiting the adhesion of these bacteria to intestinal epithelium (Ganner and Schatzmayr, 2012; Song et al., 2014). On the other hand, β-glucans have been reported to promote effects on human and animal health such as anti-tumor, anti-diabetes and anti-infection lowering cholesterol and stimulating immune properties (Du et al., 2014). This polysaccharide is also considered as the main binders of mycotoxins (Yiannikouris et al., 2006; Zoghi et al., 2014). Chitin is a fibrous polymer endowed wide interesting technological properties useful for cosmetic and medical applications (Rinaudo, 2006). However, yeast cannot be considered as commercial source for chitin production due to the its low abundance in cell wall (<1%), even though this low amount is important for the yeast cell wall properties (Aguilar-Uscanga and Francois, 2003). As for instance, we showed that cross-linkage between chitin and β-glucans is an important parameter that determines the nanomechanical properties of the yeast cell wall (Dague et al., 2010). To sum up, the yeast cell wall is endowed with several original properties relevant for various applications in health, food and feed nutrition and food safety (reviewed in Chen and Seviour, 2007; Kogan et al., 2008; Braconi et al., 2011; Pfliegler et al., 2015). However, understanding how the biochemical composition of the cell wall dictates its nanomechanical properties and unraveling molecular cues (i.e., genes) that are underlying these relationships could represent a major scientific advance toward a better mining and exploitation of technological properties of the yeast cell wall at an industrial level.

In this work, we used an integrative approach that combined genome scale expression datasets and cell wall biochemical and biophysical measurements from four different industrial yeast strains and a laboratory strain to search for relationships between genes expression, cell wall composition and cell surface characteristics. To this end, quantitative data on cell wall composition measurements (i.e., mannans, chitin, β-1,3-glucans, β-1,6-glucans), quantitative data on biophysical properties (i.e., cell wall elasticity, occurrence of the interaction with concanavalin A at the cell surface, length of the molecule unfolded) and transcriptome profiles of these 5 yeast strains were obtained. These datasets were incorporated into the mixOmics package (http://mixomics.org/; Gonzalez et al., 2009; Le Cao et al., 2009a) that integrates multiples biological data with the aim to identify variables that are highly correlated, leading to either biological inferring explanation or pertinent biological hypotheses.

## Materials and methods

### Strains and growth conditions

Four diploid *Saccharomyces cerevisiae* strains named L71, L69, L62, and L60 were provided by Lallemand Inc. (Montréal, Canada). The strain *Saccharomyces cerevisiae* BY4743 (MAT**a**/α *his3*Δ*1/his3*Δ*1 leu2*Δ*0/leu2*Δ*0 LYS2/lys2*Δ*0 met15*Δ*0/MET15 ura3*Δ*0/ura3*Δ) was obtained from Euroscarf collection. Unless otherwise stated, yeast cells were cultivated at 30°C in 1 L Erlenmeyer flask containing a 200 ml of YPD medium (2% w/v glucose, 1% w/v peptone, 1% w/v yeast extract) in a rotary shaker set at 200 rpm.

### Cell wall isolation and quantification of polysaccharides

At least three independent cultures were carried out in 200 ml of YPD as described above. Cells were collected during the exponential phase at OD_600_ of around 1.0 (correspond to 2 × 10^7^ cells/ml). Cells were harvested by centrifugation 10 min at 3,000 g and washed two times with sterile water. The pellets were kept to isolate cell walls. Cell walls were isolated and purified by centrifugation and extensive washing as described in Francois (2006). Polysaccharides mannans, chitin, β-1,3- and β-1,6-glucans in the purified cell walls were determined as described in Schiavone et al. (2014). Quantification of the released monomers (mannose, glucose and N-acetylglucosamine) was determined by high performance anionic exchange chromatography (HPAEC) coupled to amperometric detection as described by Dallies et al. (1998), and colorimetric method, respectively (Reissig et al., 1955).

### Determination of hydrophobicity

Hydrophobic properties of yeasts strains were determined by measuring their affinity for an apolar solvent as described in Purevdorj-Gage et al. (2007). Briefly, overnight cultures were centrifuged at 2,000 g for 5 min and resuspended in fresh YPD medium to obtain an optical density of 1 at 600 nm. After 3 h of incubation at room temperature, the OD_600_ was measured for each culture. In 15 × 100 mm borosilicate glass tubes 0.6 ml of octane (Sigma-Aldrich) was added to 1.2 ml of yeast cell suspension. The mixtures were vortexed for 120 s and allowed to stand for 15 min at room temperature to achieve the complete separation of the two phases. The aqueous phase was recovered and its OD_600_ measured. The results were expressed as the Octane adhesion index, (% A) which represents the percentage of cells retained by the organic fraction, according to the relationship: % A = [A_0_-A_F_/A_F_] × 100. A_0_ and A_F_ represent the optical density at 600 nm of the yeast suspension before and after contact with octane. Assays were performed in triplicates.

### AFM measurements

#### Sample preparation

Strains were stocked at −80°C, revivified on Yeast Peptone Dextrose agar (from Difco) and grown in 5 mL of YPD at 30°C i 15 ml culture tubes shaken at 200 rpm. Yeasts cells were collected at the exponential phase (OD_600_ ~ 1), washed two times in acetate buffer (18 mM sodium acetate, 1 mM CaCl_2_, 1 mM MnCl_2_, pH = 5.2), resuspended in the same buffer, and immobilized on polydimethylsiloxane (PDMS) stamps prepared as described in Formosa et al. (2015b). Briefly, freshly oxygen activated microstructured PDMS stamps were covered 100 μL of the solution of cells. The cells were then introduced into the microstructures of the stamp by convective/capillary assembly.

#### AFM imaging and force spectroscopy

Images and force-distance curves were recorded at room temperature in a 50 mM acetate buffer pH 5.5 using an AFM Nanowizard III (JPK Instruments, Berlin, Germany) and MLCT AUWH cantilevers (Brüker, Santa Barbara, USA). The spring constants of the cantilevers were systematically measured by the thermal noise method (Hutter and Bechhoefer, 1993) and were found to be in the range of 0.01–0.02 N.m^−1^.

Images were recorded in Quantitative Imaging™ mode (Chopinet et al., 2013) with a maximal applied force of 1.5 nN and approach speed of 12 μm.s^−1^. Mechanical properties were mapped by recording an array of 32 × 32 force-distance curves using a maximal applied force of 0.5 nN and a speed of approach and retraction of 2 μm.s^−1^, corresponding to loading rates ranging from 20,000 to 40,000 pN.s^−1^. Elasticity histograms were generated by analyzing with OpenFovea software (Roduit et al., 2012) the force (F) curves according to the Hertz model with an indentation (δ) of 50 nm and taking into account a conical tip geometry with an half-opening angle α of 0.31 rad and a Poisson ratio (ν) of 0.5:

$$\begin{array}{l} F=\frac{2E\tan\alpha }{\pi \;\left(1-\nu^{2}\right)}.\;\delta^{2} \end{array}$$

The Young's (or Elasticity) modulus is the value obtained at the maximal height of the Gaussian curve ± σ value (corresponding to the mid height width) of this Gaussian distribution function. Prior to force spectroscopy experiments, AFM tips were functionalized with the lectin concanavalin A from *Canavalia ensiformis* (ConA; Sigma-Aldrich) via a dendritip as described in Jauvert et al. (2012). The coupling with the lectin was made by immersion of the dendritip in 100 μL of ConA solution (100 μg.mL^−1^ in 0.1 M sodium carbonate buffer). After 1 h of incubation, 100 μL of NaBH_4_ (3.5 mg.mL^−1^) solution was added and incubated 15 min in order to reduce the unreacted groups. The cantilever was washed three times in acetate buffer and immediately used. Using the functionalized tip, force-distance curves were recorded on each yeast cell with a maximal applied force of 250 pN, and using a constant approach and retraction speed of 2 μm.s^−1^. At least 8 cells of two independent cultures were analyzed for each strain, representing 8,192 force curves. All force curves were analyzed with the JPK Data processing software. All specific adhesion peaks were considered for the histograms, which were generated using Origin 8 software (OriginLab Northampton, MA, USA). The extension of polysaccharides at the surface of the cell with the AFM-tip functionalized with ConA was analyzed using either the freely-jointed chain (FJC) model for single adhesion event or the worm like chain (WLC) model for multiple adhesion events. The WLC model introduced by Bustamante et al. (1994) describes the polymer chain as a curved filament and the force F vs. the extension (or distance) × is given by:

$$\begin{array}{l} \text{F}\left(\text{x}\right)={\text{k}}_{\text{b}}\text{T}/{\text{l}}_{\text{p}}\left[0.25{\left(1-\text{x}/{\text{L}}_{\text{c}}\right)}^{-2}+\text{x}/{\text{L}}_{\text{c}}-0.25\right] \end{array}$$

Where k_b_ is the Boltzmann constant, T the absolute temperature, l_p_ is the persistent length and L_c_ is the contour length. The persistent length gives information about the degree of structural rigidity of the polymer chain and is defined as the longest segment below which the chain can be considered as a rigid rod, the extension of the polymer until the point at which the force necessary to extend further and in our case to break the interaction with the functionalized AFM—tip corresponds to the contour length. In the FJC model, the polymer is modeled as a chain of equal, independent and freely rotating segments (Rief et al., 1997). The model describes the elastic behavior of a polymer with three adjustable parameters: the contour length l_c_, the Kuhn length l_k_ which is the length of a segment and is a direct measure of the chain stiffness, and the elasticity of the segments *K*_s_. The extension x vs. the pulling force (or adhesion force) is given by the following equation:

$$\begin{array}{l} \text{x}\left(\text{F}\right)={\text{L}}_{\text{c}}\left[\text{coth}\left(\text{F}{\text{l}}_{\text{k}}/{\text{k}}_{\text{b}}\text{T}\right)-{\text{k}}_{\text{b}}\text{T}/\text{F}{\text{l}}_{\text{k}}\right] \end{array}$$

where F is the extension force (N), x is the extension of the polymer (m), k_b_ is the Boltzmann constant and T the absolute temperature. The contour length L_c_ for n segments is defined as: L_c_ = nl_k_.

### DNA microarray analysis

For each yeast strains, three independent cultures were carried out in 50 ml of YPD in a 250 mL shake flasks on a rotary shaker set at 200 rpm at 30°C. Yeast cells (a total of about 10 OD_600_ units) were collected at OD_600_ at about 1.0 by centrifugation (3,000 rpm, 4°C, 2 min), followed by a washing step with 1 mL of sterilized water. The cell pellets were immediately frozen in liquid nitrogen and stored at −80°C until RNA extraction.

#### Total RNA extraction

Frozen cells were mechanically disrupted using a ball mill (MicroDismembrator Braun, Melsungen, Germany). Total RNA was extracted using SV Total RNA Isolation System (Promega) following the protocol of the manufacturer. The quantity of extracted RNA was controlled using Nanodrop ND-1000 (Nanodrop Technologies) and the quality was determined by microcapillarity electrophoresis using a Bioanalyzer 2100 (Agilent Technologies, Wilmington, USA). Incorporation of Cyanine 3 was performed during reverse transcription of total RNA using the Low Input Amp Labeling kit (Agilent Technologies, Wilmington, USA).

#### DNA microarray

The One-Color Microarray-Based Gene Expression Analysis Protocol was carried using. labeled cDNA which was purified and hybridized on Agilent glass slides that bear the whole *Saccharomyces cerevisiae* genome (see details at http://www.biocompare.com/ProductDetails/760330/S-cerevisiae-Saccharomycescerevisiae-Whole-Genome.html). Hybridization was carried out in an automatic hybridization chamber (Agilent Technologies, Wilmington, USA) for 17 h at 65°C. The hybridization signals were detected by scanning using Innoscan 900 laser Scanner (Innopsys Instruments), and transformed to numerical values using Feature Extraction V.11.5.1.1. The microarrays hybridization and processing were carried out at the Transcriptome-Biochips Platform of Toulouse (http://biopuce.insa-toulouse.fr). The microarray work is fully MIAME-compliant and the data have been deposited in the Gene Expression Omnibus (GEO) Database (http://www.ncbi.nlm.nih.gov/geo/query/acc.cgi?acc=GSE78759), under the accession number GSE103392.

#### Data analysis

Transcriptome analyses were done in R computing environment (R Core at https://www.R-project.org/) using the Limma package available on the R repository Bioconductor (www.bioconductor.org). The estimates used for the foreground and background intensities were the median of pixels intensity. Raw data were imported into R and spot quality weights were performed assigning a weight of 1 or 0 to each spot. Low-quality spots, non-uniform spots, spots with low signal/background ratio or spots with low signal-to-noise ratio and empty or non-validated spots were down weighted. Data were preprocessed by base 2 logarithmic transformation and within-array normalized was performed using the weighted global median (spots with zero weight were not included in the normalization). To achieve consistency of expression values between arrays, quantile normalization across all the microarrays for each strain was performed. After normalization, the expression of a gene was calculated by the median of replicate spots within each microarray. Gene expression data for both strains were pairwise compared using the Limma package (Smyth, 2005). Genes with significant evidence for differential expression were identified with a modified *T*-test in conjunction with an empirical Bayes method to moderate the standard errors of the estimated log-fold changes. The *p*-values were adjusted for multiple testing by the “BH” method (Hochberg and Benjamini, 1990).

The microarray work is fully MIAME-compliant and the data have been deposited in the Gene Expression Omnibus (GEO) Database (http://www.ncbi.nlm.nih.gov/geo/query/acc.cgi?acc=GSE78759), under the accession number GSE103392.

### Relationships between gene expression, biochemical and biophysical data

The package mixOmics employs partial least square (PLS) based methods to unravel relationships between variables from multiple biological datasets. The tool has its own tutorial and can be easily used, for a nearly inexperienced R user, through the web site at http://mixomics.org/. Specific functions were searched on the *Saccharomyces* Genome Database (http://www.yeastgenome.org), Yeastract (http://www.yeastract.com/) and Funspec (http://funspec.med.utoronto.ca/).

## Results

### Yeast strain-dependency of biochemical composition and nanomechanical properties of the cell wall

Biochemical composition of cell wall was determined in the four industrial and the laboratory strains cultivated that were harvested in exponential phase (i.e., OD_600_ ~1 unit) of growth on a glucose rich medium. Table 1 recapitulates the data obtained for the three polysaccharides that constitute the yeast cell wall. It can be see that the mannans content in cell wall of three out of the 4 industrial strains was 30% higher than in BY4743 strain (laboratory strain). This higher mannans content in strains L60 and L69 was actually expected since these strains are wine yeasts, whereas L20 has been selected after mutagenesis for higher mannoproteins content (Lallemand Inc., personal communication). In correlation with this high content of mannans, the hydrophobicity of these three strains was 3–6-times higher than that of the lab strain. However, this property is likely not entirely associated with levels of mannans since the industrial strain L71 exhibited a similar hydrophobicity as L62 and L60 strain albeit the proportion of mannans in L71 strain was comparable to that of the laboratory strain BY4743. An additional relevant data that reveals strain-dependency of the cell wall composition is concerning the β-glucans content and more specifically the β-1,3-glucans/β-1,6-glucans ratio. In the industrial strains L71, L62, and L60, the proportion of β-1,6-glucans reached more than 50% of total β-glucans, whereas it represented less than 30% in the lab strain, in accordance with a previous report (Schiavone et al., 2014). Whether this higher proportion of β-1,6-glucans in industrial strains in spite of lower amount of total β-glucan in these strains as compared to the lab strain can explain the remarkable efficacy of these industrial strains to stimulate the immune response (Lallemand Inc., unpublished data) remains to be verified. Finally, the proportion of chitin in cell wall was found around 4% in all strains, which agrees with previous works (Schiavone et al., 2014), with the exception of L62 strain for which it was 2 times higher. Collectively, these results showed high variability in the composition of the cell between strains, which may result from the selection and adaptation of yeasts to specific cultures/process conditions.

**Table 1:** Summary of biochemical and biophysics data of a laboratory and four industrial *Saccharomyces cerevisiae* strains.

**Strain**	**Chitin**	**β-1,3-glucans**	**β-1,6 glucans**	**Mannan**	**Hydrophobicity (%)**	**Stiffness (kPa)**	**Adhesion event (%)**	**Contour length (nm)**	**Size (μm)**
BY4743	4.3 ± 0.8	44.7 ± 2.9	19.5 ± 1.3	33.5 ± 2.8	7.8 ± 4.2	483 ± 61	8 ± 1.5	64.8 ± 30	3.8 ± 0.7
L71	5.7 ± 1.1	30.8 ± 2.20	29.4 ± 1.7	32.3 ± 1.3	25.1 ± 5.3	637 ± 178	25 ± 6	20 ± 14.7	5.0 ± 1.2
L62	9.9 ± 1.1	20.4 ± 1.9	23.1 ± 2.7	46.5 ± 1.3	23.8 ± 9.7	239 ± 52	33 ± 4	52.6 ± 13	3.8 ± 0.9
L60	4.5 ± 0.7	19.6 ± 5.4	24.1 ± 4.7	46.5 ± 3.7	23.1 ± 6.8	438 ± 64	28 ± 4	25.1 ± 4.3	4.4 ± 1.0
L69	3.9 ± 1.0	34.1 ± 3.6	18.8 ± 3.1	43.5 ± 2.5	45.6 ± 5.7	230 ± 82	21 ± 2	96.9 ± 8.3	4.2 ± 1.1

*Biochemical (chitin, β-glucans, mannan) and biophysics (hydrophobicity, stiffness, Force of adhesion, adhesion events and contour length) variables were determined on exponentially growing yeast cells in YPD as described in Material and Methods. Chitin, β-1,3-glucans, β-1,6 glucans and mannans are expressed as % of total polysaccharides. The value reported are the mean values ± SD obtained from at least three biological independent experiments*.

**Figure 1:**
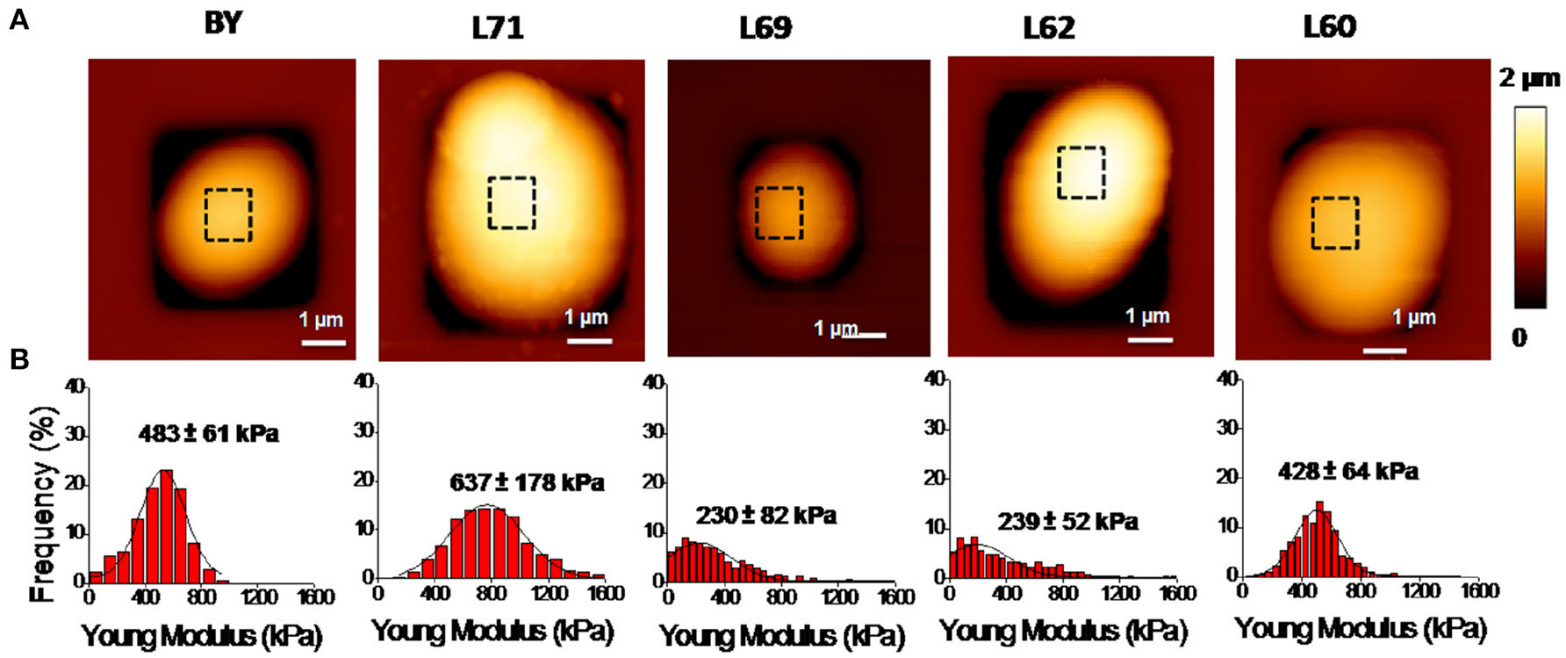
AFM imaging and elasticity property of the laboratory strain BY4743 and 4 industrial *Saccharomyces cerevisiae* strains. **(A)** Shows a high resolution AFM image (z-range = 100 nm; scale bar = 0.20 μm) of an exponentially growing cell of the laboratory strain BY4743 and of the four industrial strains. **(B)** Shows the distribution of the Young's modulus generated from 1,024 force curves from which is determined the maximal value as described in section Material and Methods.

To investigate on a potential association between the biochemical composition and the nanomechanics of the cell wall, we determined the elasticity (Young) modulus by performing nano-indentations experiments in which the deformation depth is plotted as a function of the force applied by the AFM silicon nitride tip on the cell. For these AFM experiments, the yeast cells were individually trapped in PDMS-fabricated microchambers according to the method described previously (Dague et al., 2011). AFM imaging of one individual cell from each strain is illustrated in Figure 1A and shows relatively identical surface topology of these yeasts. For elasticity modulus, the AFM measurements were performed on areas devoid of bud scars and on at least 5 independent cells for statistical analysis of the data. As described in section Materiel and Methods, 1,024 indentation curves are performed on each cell and fitted to the Hertz model. This generates histograms of Young's modulus value that can be adjusted to a Gaussian distribution The most probable value of the Young's modulus corresponding to the maximal height of the Gaussian curve gives a quantitative indication of the cell stiffness. It is shown in Figure 1B that this value is remarkably different between strains and hardly difficult to correlate with the biochemical composition of the cell wall (Table 1). Notably, strain L62 which contains twice more chitin in the cell wall than the four other strains exhibited the lowest Young's modulus value of 239 kPa, whereas it was previously reported that the chitin level is important for the stiffness of the cells (Touhami et al., 2003). On the other hand, the industrial strain L60 and lab strain have a comparable elasticity modulus, but the content in β-glucans and mannans in these two strains is completely different. Taken together, these data reinforce our earlier work showing that the elasticity property of a yeast cell is independent on its cell wall composition (Dague et al., 2010; Francois et al., 2013).

### Probing cell surface of yeast strains using AFM tips functionalized with concanavalin A

**Figure 2:**
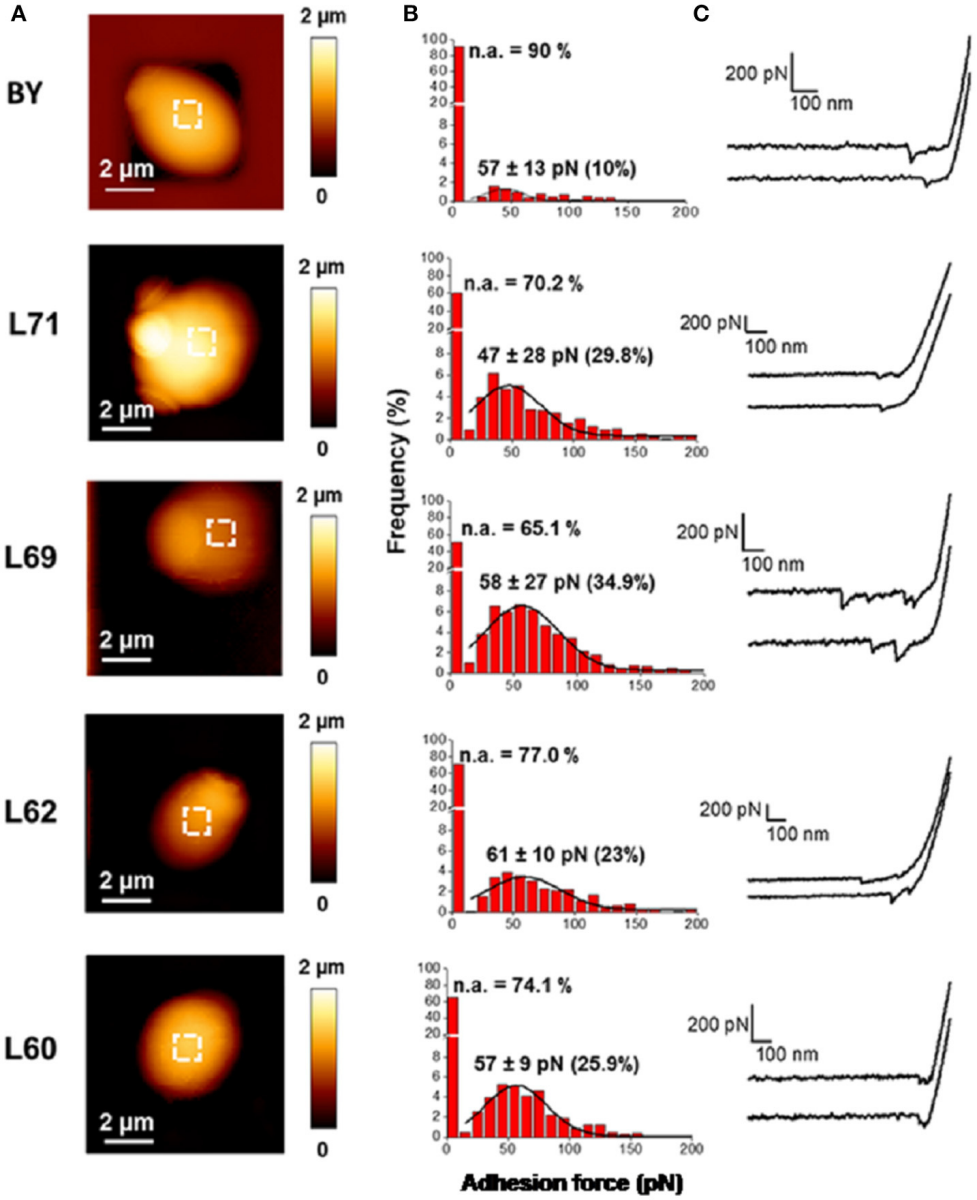
Probing cell wall architecture of the laboratory strain and 4 industrial strains with AFM tips functionalized with concanavalin A. AFM height **(A)** images of the laboratory and industrial strains are shown. Adhesion forces using AFM tips functionalized with ConA **(B)** were obtained with 8 cells from 2 independent experiments (8,192 curves were analyzed with JPK data processing). The data are presented as frequency (in %) of adhesion event vs. adhesion force. In **(C)** is illustrated two representative force curves recorded with the AFM-tip ConA for each strain.

The difference in hydrophobicity between industrial and lab strains which is at first glance not directly associated with higher levels of mannans is suggestive of some dissimilarity in the organization, structure or degree of mannosylation of cell wall proteins between these strains. To investigate this question, we employed single molecule force spectroscopy (SMFS) using AFM-tips functionalized with concanavalin A (AFM tip-ConA), a plant lectin known to bind specifically α-mannose residues of glycoproteins (So and Goldstein, 1968; Gad et al., 1997). This technique allows to probe the distribution, adhesion, flexibility and extension of mannoproteins at the surface of yeast cells. Figure 2A shows that the surface topology of these strains as recorded in Quantitative Imaging™ mode (Chopinet et al., 2013) was very comparable. However, the adhesion frequency of functionalized tip at the cell surface vs. the force needed to break the interaction (unbinding force) was only 10% for strain BY while these adhesion events were in the range of 25–35% with these industrial strains. Thus, these data indicated that the cell surface of industrial strain is physically distinct from that of the lab strain. In spite of this difference, the adhesion force which also corresponds to the force needed to unbind the interaction of the tip-ConA with the cell surface was estimated in the range of 55–60 pN (Figure 2B). This value is very similar to the one obtained in previous works (Alsteens et al., 2008; Francius et al., 2009) using AFM tip functionalized with ConA through a 6 nm long polyethylene glycol (PEG) chain. This data indicates that the unbinding force for a single lectin—α-mannose interaction is not modified by the type of spacer between the tip and the ConA protein. Finally, we confirmed that the AFM-tip ConA directly interacts with cell wall mannoproteins since the interaction was almost complete lost by adding 100 mM mannose prior to the AFM measurements (data not shown). Figure 2C also shows that single adhesion events take place as deduced from the pattern of force-distance curves obtained with AFM-tip ConA, except for L69 strain which revealed more than one event. Therefore, for strains BY4743, L71, L62, L60, the unbinding force of the single adhesion event could fit a freely jointed chain (FJC) model with a Kuhn length (*k*_l_) in the range of 0.05–1 nm (see Figure S1). For L69 strain, the multiple unbinding peaks were well fitted with a WLC model with persistent lengths (Ip) ranging from 0.025 to 2 nm. This high variation of this parameter suggested a relative high flexibility of the mannans chains or mannoproteins pulled by the AFM tip.

**Figure 3:**
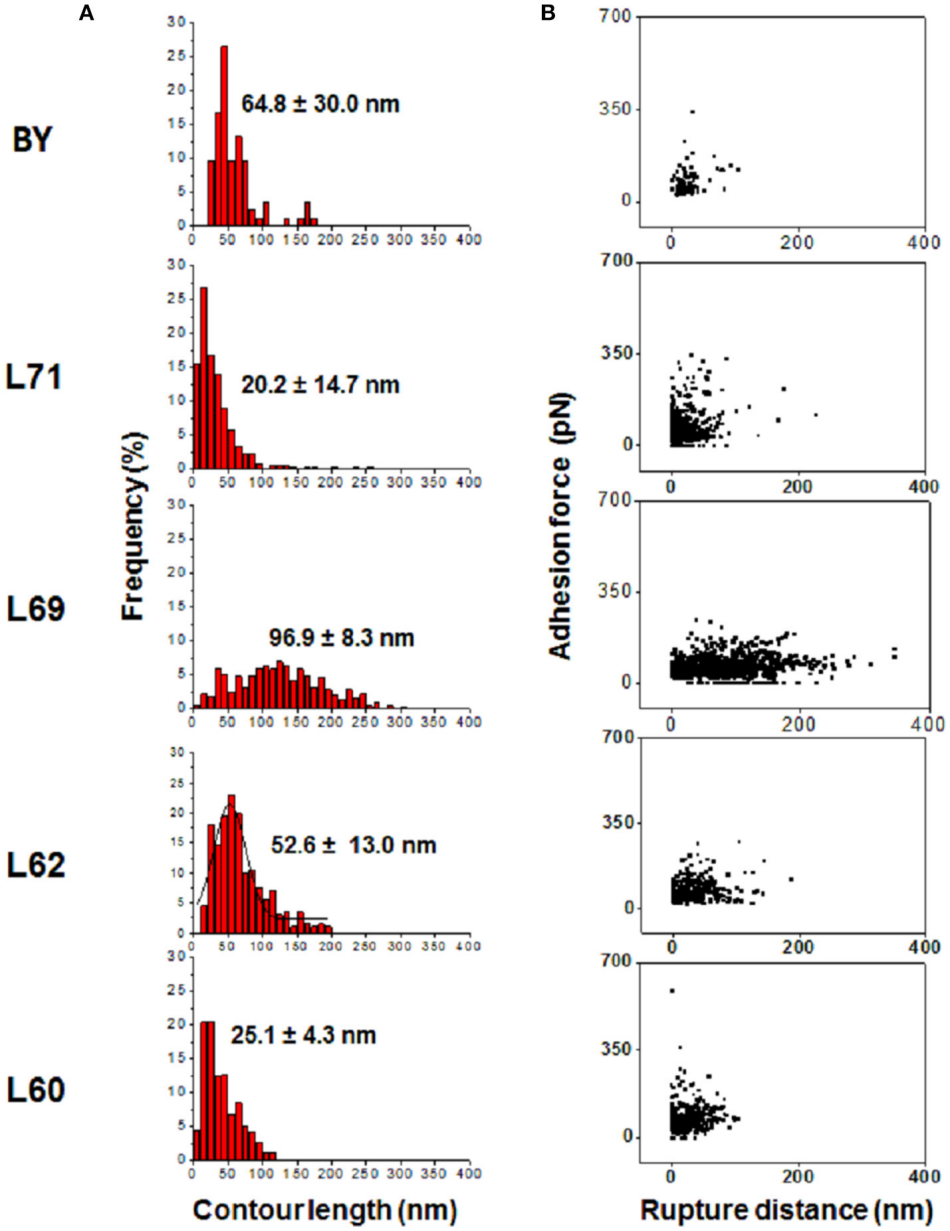
Comparison of contour lengths and rupture distances between strains. **(A)** Shows the distribution of contour length determined from 8,182 forces-distance curves after retraction of the AFM tip-ConA before the rupture. In **(B)** is reported the plots of the 8,182 adhesion force vs. rupture distance obtained for each of the 5 strains with the AFM tip-ConA.

Use of the FJC and WLC models allowed to determine the contour length, which correspond to the extension or relaxation of mannans chains obtained by pulling with the AFM tip-ConA at low force until the force necessary to extend further rises rapidly and ends up by breaking the binding (Fisher et al., 1999). As illustrated in Figure 3A, the contour length values roughly followed a Gaussian distribution, with the shape of the curve and the mean value not the same between the strains, suggesting some structural differences of the cell wall mannans/mannoproteins in these strains. Except with strain 69 for which the contour length distribution ranged from 20 to 300 nm, with a mean value around 97 nm, the distribution was sharp with a mean value centered at 20–25 nm for strain L71 and L60, and was 2.5 times higher in strains L62 and BY4743. An additional pertinent physical data that can be obtained from the adhesion force vs. distance curves is the rupture distance (Figure 3B) which corresponds to the distance at which the interaction of ConA with the mannosyl unit of the mannoproteins is broken, i.e., when the adhesion force is reset to zero (see Figure S1). Interestingly, strain L69 that showed the longest contour length also presented the longest rupture distances that could reach up to 400 nm. However, this relationship between contour length and rupture distance was not respected in the other strains since the rupture distances in strain 71 were about 2 times higher than in strain 60 albeit both strains exhibited similar contour lengths (Figure 3B). A similar statement could be made for strain BY4743 and L62. Altogether, these data highlighted significant differences in the physical properties of cell wall mannans between yeast strains.

### Overview of transcriptomic profiles of industrial strains relative to the laboratory strain

The biochemical and biophysical data reported above raised the question whether these differences can be in part related to changes in genes expression between strains. We addressed this question by exploring and comparing the transcriptome of industrial and laboratory strains using DNA microarrays technology. As a first step, we analyzed the global transcriptomic data from three biological replicates of each strain using Partial least square (PLS) regression analysis developed elsewhere (Le Cao et al., 2008) as this method can highlight similarities and dissimilarities between samples. This analysis clearly denoted the good repeatability of the transcriptome analysis for each strain (Figure S2). It also illustrated that the transcriptomes of the 4 industrial strains are relatively close and in particular those of strains L60 and L62 which are very neighbors, whereas the transcriptome of the diploid lab strain significantly separates from the four industrial yeasts. Looking for genes that were differentially expressed in the industrial strains relative to the lab strain BY4743, lists of 162, 210, 190, and 166 genes in L71, L69, L62, and L60 were retained, taking as criteria only genes that were differentially expressed by a factor ≥2 at a *p*-value < 0.01. Venn representation of these data showed that 10–25% of the differentially expressed genes were strain-specific and among these lists, a set of 71 genes emerged as being differentially expressed in all four industrial strains relative to the lab strain (Figure S3 and Table S1 for detailed description of the gene function).

**Figure 4:**
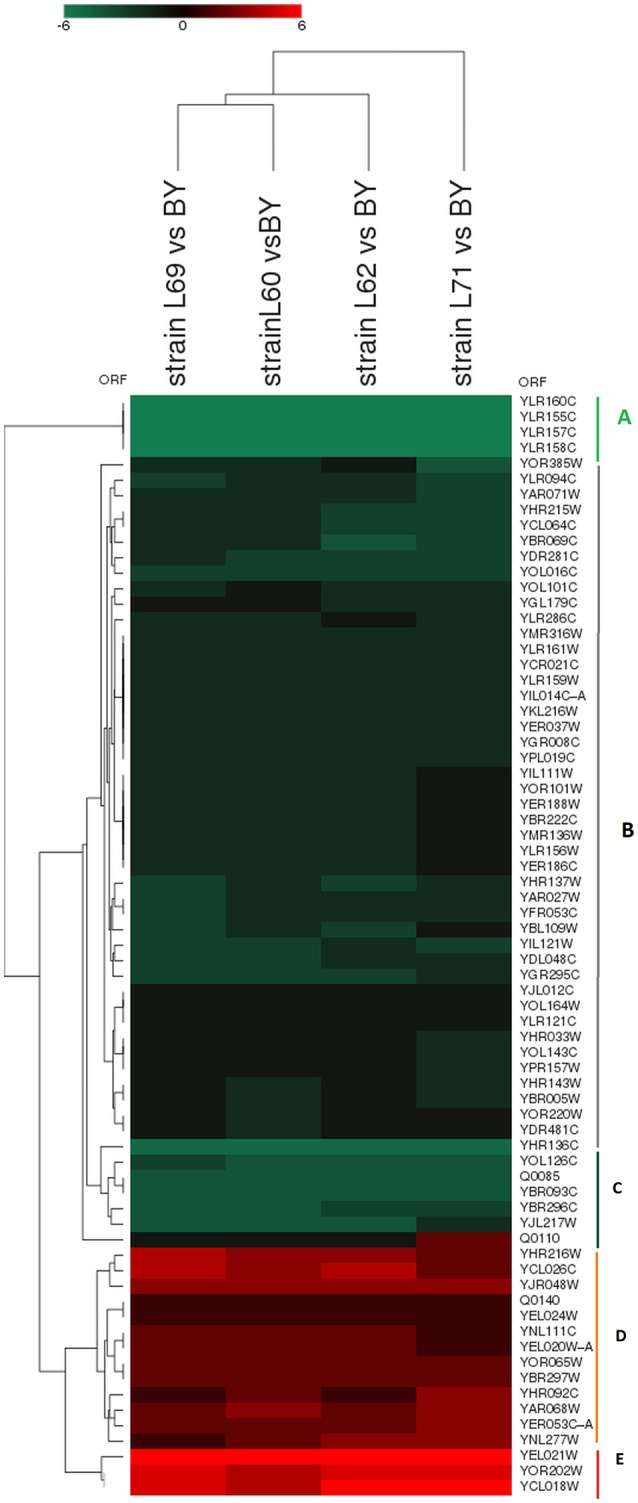
2D-clustering analysis of the 71 common genes that are differentially expressed in the 4 industrial yeasts vs. laboratory strain BY4743. Functional analysis of the sub-group (**A**, 4 genes), (**B**, 46 genes), (**C**, 6 genes), (**D**, 12 genes), and (**E**, 3 genes) are described in the text and in Table S1.

Functional classification of these 71 genes into GO-molecular function and GO-cellular component showed enrichment of cell wall-bounded enzymes which included by genes encoding acid phosphatases and cell wall associated L-asparaginases and in mitochondrial function including heme binding and electron transport activity (Figure S4). A 2D-cluster analysis of this genes list could be further broken down into 5 subgroups (Figures 4A–E). The subgroup A included only 4 genes (*YLR155C/ASP3-1, YLR157C/ASP3-2, YLR158C/ASP3-3*, and *YLR160C/ASP3-4)* that encode the cell-wall or periplasmic localized L-asparaginases. The strong downregulation of *ASP* genes family in these four industrial strains are in line with previous results indicating their absence in the genome of 128 fungal species including wine and brewing yeasts (Pope et al., 2007; Carreto et al., 2008). Conversely, the subgroup E comprises *LEU2, HIS3*, and *URA3* whose expression is extremely high in industrial yeasts because these 3 genes are used as auxotrophic marker in the laboratory strain and thus are transcriptionally defective. A large subgroup (B) of about 45 genes showed a slight (1.5) to moderate (3–4-fold) lower expression in industrial strains as compared to laboratory strain. Main GO biological processes in this group were related to phosphate metabolic process such as *YHR215w*/*PHO12, YAR071w*/*PHO11, YDR281c*/*PHM6*, and *YERO57w/PHM8* whose expression is known to be induced by low P_i_ or repressed by high P_i_ (Ogawa et al., 2000). This data suggests that industrial strains may have a higher bioavailability of inorganic phosphate. This inferred “higher Pi content” in these strains is further illustrated in the subgroup C in which *YBR093C/PHO5* encoding the major phosphate repressible acid phosphatase (Huang and O'Shea, 2005), the high affinity Na+/Pi cotransporter encoded *PHO89/ YBR296c* gene (Auesukaree et al., 2003) and *SLP2* encoding a protein that targets the low affinity H^+^/Pi transporter encoded by *PHO87* into vacuole for degradation (Ghillebert et al., 2011) were 10–20-fold less expressed than in the laboratory strain. Finally, the subgroup D comprised a short list of 12 slightly to moderately upregulated genes in industrial strains (i.e., from 1.5 to 4-fold more expressed) whose mitochondrial electron transport chain and mitochondrial respiratory complex III were identified as the main GO biological process and GO-component, respectively by the Funspec (http://funspec.med.utoronto.ca/) and GO term finder tools (http://www.yeastgenome.org/cgi-bin/GO/goTermFinder.pl). More specifically, upregulation of the *RIP1* and *CYT1* encoding two of the three catalytic subunits of the cytochrome bc1 and of *CYC1* encoding cytochrome C1 suggests that, even under high glucose condition for which respiration is strongly repressed but very likely not completely shut off (Lagunas, 1986), the industrial strains may acquire more energy from the oxidative phosphorylation than the lab strain since cytochrome bc1 is critically important for this function (Hunte et al., 2003). Alternatively, the apparent higher respiratory activity of the industrial strains could be an indirect effect of the well-known poor respiratory competency of the laboratory strain BY4743 that is derived from S288c background (Young and Court, 2008).

### Most of the differentially expressed genes in industrial strains relative to the laboratory strain are implicated in cell wall function

**Figure 5:**
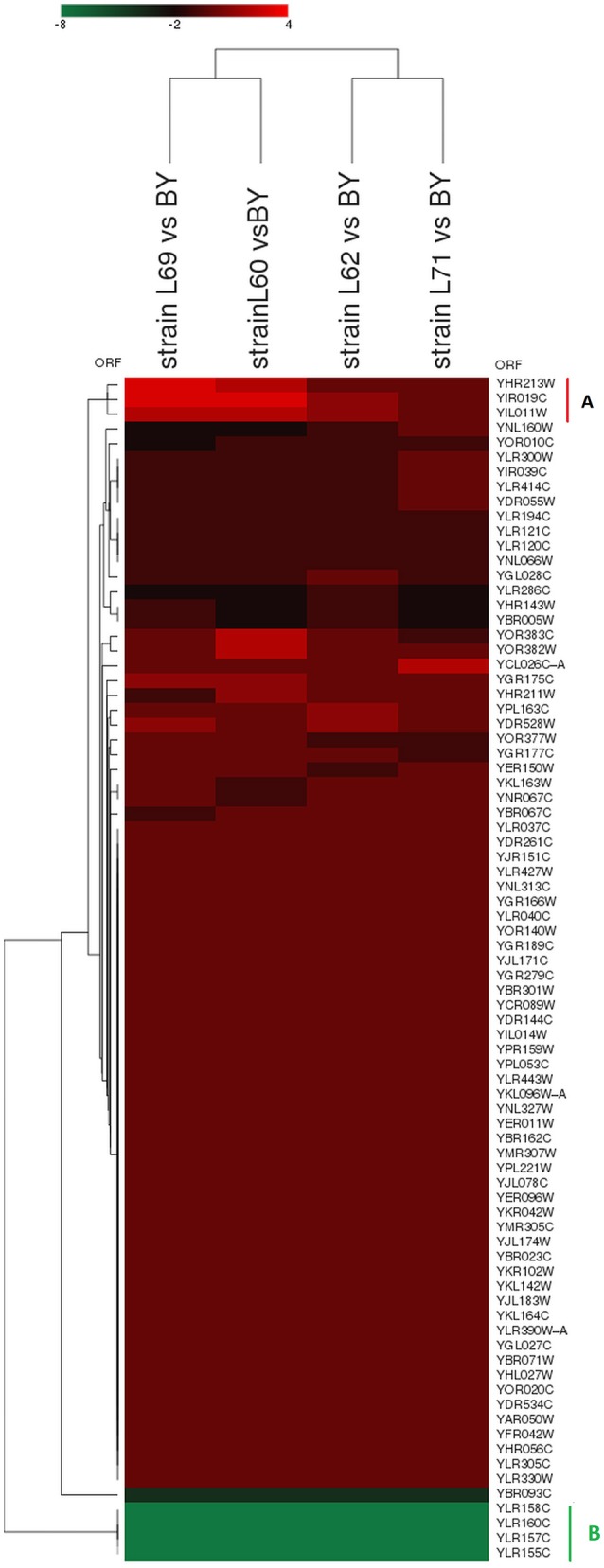
2D-clustering analysis of differentially expressed genes related to cell wall organization between the four industrial strains and the laboratory strain. The sub-group **(A)** contains the most highly upregulated genes, with*YHR213*w encoding a pseudogene homologs to *FLO1, YIR019*c encoding a Flo11 adhesin and *YIL011w* (*TIR1*) encoding a cell wall serine-alanine rich protein. The subgroup **(B)** correspond to the Yapsin genes that are not expressed in industrial yeasts.

The transcriptome profile of the 4 industrial yeast strains compared to that of the laboratory strain BY4743 revealed a remarkable enrichment of genes implicated in the cell wall architecture and remodeling. Thus, a total of 80 genes differentially expressed between industrial and laboratory strains was retrieved and subjected to a detailed functional analysis (see Table S2 for detailed description of the gene function). A 2D-clustering representation of these differentially expressed genes showed that, apart from the *ASP* genes family which are strongly downregulated likely because they are absent in these industrial strains (see above), most of the other cell wall related genes were broadly upregulated but to different extent in the 4 industrial strains (Figure 5 and see Figure S5 for G0 biological and molecular function classification). In particular, we noticed a potent upregulation of the adhesin-encoded *FLO11/YIR019C* and of *YHR213w* which codes for a protein sharing sequence similarity with the adhesin-encoded *FLO1* gene (Teunissen and Steensma, 1995) in strain L69 and L60. Interestingly, this upregulation of these two genes which was the highest in strain L69 was concomitant with the highest hydrophobicity displayed by this strain (Table 1) and with the observation that this strain formed large aggregates in growth on glucose (data not shown). On the other hand, the upregulation of several genes (*YIL011w/TIR3; YOR382w/FIT2; YOR383c/FIT3, and YER011w/TIR1*) encoding glycosylphosphatidylinositol (GPI) anchored cell wall mannoproteins in strain L69, L62, and L60 could explain their higher content of mannans in cell wall (Table 1), whereas the downregulation of 3 out of the 5 Yapsin family genes shown to be involved in glucans homeostasis (Krysan et al., 2005) could account for lower β-glucans in these industrial yeasts as compared to lab strain. This transcriptomic change together with the slight upregulation (i.e., 1.5–2.5) of *KTR6* implicated in mannosylation of proteins (Lussier et al., 1997), *KEG1* involved in β-1,6 glucans synthesis (Nakamata et al., 2007), *HLR1* and *EMW1* encoding proteins implicated in cell wall maintenance and integrity (Versele and Thevelein, 2001) may contribute to a wall composition and structure of the industrial strains that differs from the laboratory strain. As a final note, we noticed a 1.6–2.2-fold increase in the expression of *ERG1* (Table S2) in the 4 industrial yeast strains. The higher expression of this gene that codes for the first enzyme in ergosterol synthesis pathway from squalene (Leber et al., 1998) might be attributed to the tendency of these strains to be cultured under microaerobic conditions. To conclude, differences in cell wall composition and architecture between industrial and laboratory strains could be grossly inferred from differential genes expression. However, this transcriptomic analysis remains only informative to unravel the molecular cues that account for the biochemical and biophysical properties of cell wall and how these two parameters are interconnected.

### Inference of genes function that correlate with cell wall biochemical and biophysical properties by multivariate methods analyses

**Figure 6:**
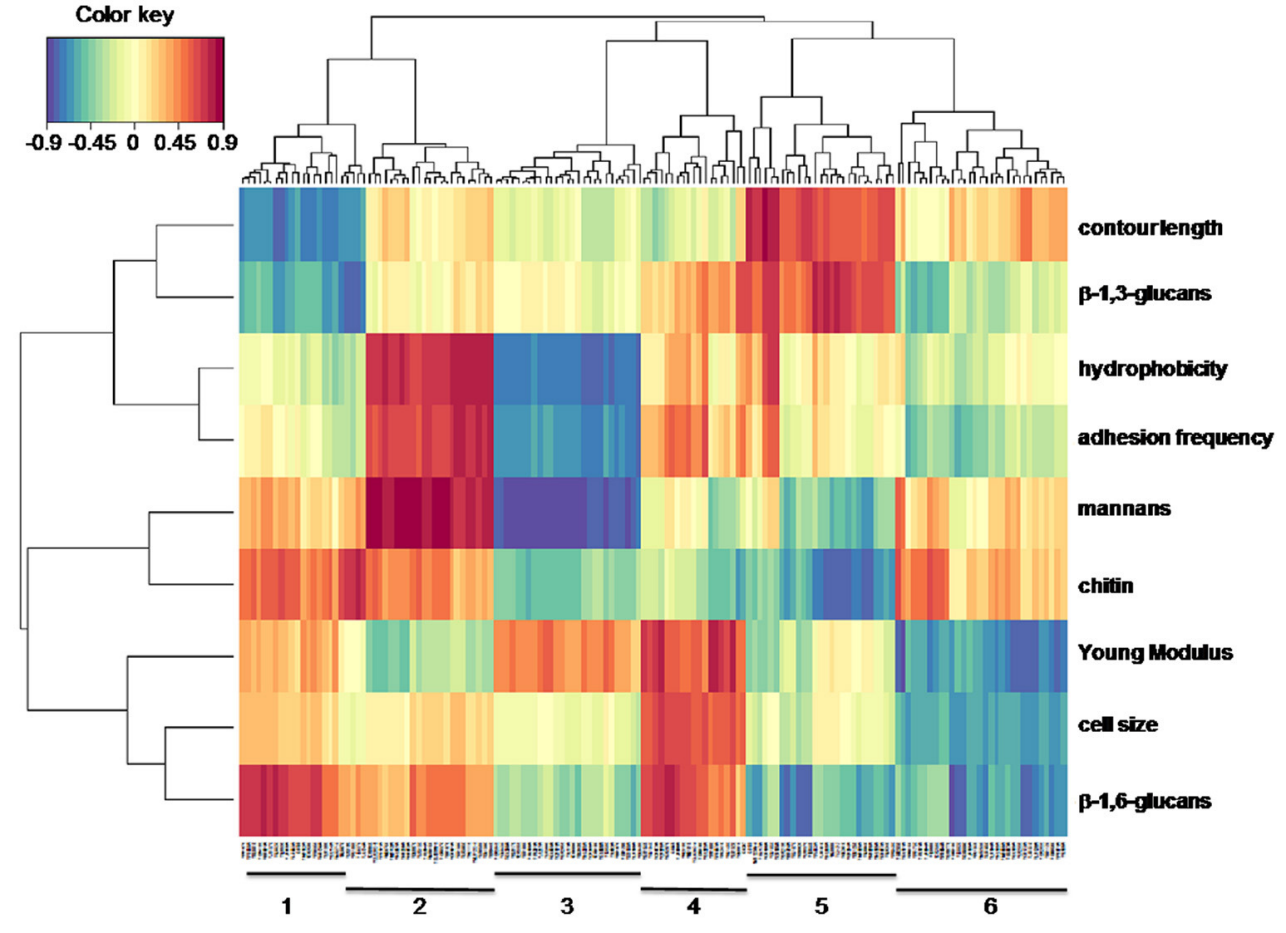
Heat map representation of association between biophysical-biochemical variables and genes transcripts from the laboratory and industrial strains. Biophysical and biochemical variables are presented on the vertical scale whereas the gene transcripts are on the horizontal scale. Clustering analysis made with the mixOmics tool (http://mixomics.org/) allows grouping biochemical and biophysical variables into 4 groups (horizontal) and into 6 groups with respect to gene transcripts (vertical). Red (blue) indicates high positive (negative) correlation between gene transcript and biochemical/biophysical variables.

The polysaccharides composition of the cell wall, Young modulus, adhesive events with concanavalin A, hydrophobicity and transcriptome are a set of biochemical, biophysics and molecular data that must be connected in one way or another. To unravel the connection between these high dimensional data where the number of variables (e.g., genes, proteins, metabolites) is much larger than the number of samples (i.e., yeast strains in this work), we employed the sparse methods of the mixOmics package. Exploration of relationships and correlation between these data sets requires reducing the dimension of the variables into components, from which can be retrieved correlations that are amenable to statistical inference about novel function or biological hypotheses by employing several *sparse* multivariate models (Gonzalez et al., 2009; Le Cao et al., 2009a). The sparse methods include a selection jointly performed with the search for correlated variables. We thus initiated our exploration analysis using the Sparse Partial least square (sPLS) regression mode which allowed to capture genes (component 1) whose the absolute expression level is the most correlated with the physico-chemical and biochemical variables (component 2). The correlation between component 1 and component 2 are projected into a correlation circle plot in which it can be seen that with, the exception of the variable “cell size,” all other parameters are located outside a 0.5 circle value, indicating either a positive (value > +0.5) or a negative (value < −0.5) correlation between them (Figure S6). According to Gonzalez et al. (2012), correlation between these variables is better visualized in a 2-dimensional heat map representation where relations between biophysical and biochemical variables are reported on the vertical axis whereas the horizontal axis shows the genes whose expression are the most correlated with these two variables (Figure 6). Confidence about this multivariate methodology sPLS is provided by retrieving predictable correlations such as between hydrophobicity and adhesion events (i.e., frequency of interaction of the concanavalin A-functionalized AFM tips on the yeast cell surface), as well as confirming that there is no simple correlation between the Young's modulus and any of the three types of cell wall polysaccharides (Dague et al., 2010; Schiavone et al., 2016). On the other hand, pertinent associations can be pointed out thanks to this statistical method such as a connection of β-1,3-glucans with the contour length or unexpected ones like the association of mannans and chitin as one would predict an association of chitin with β-1,6 glucans according to the cell wall structure proposed elsewhere (Klis, 1994).

On the horizontal axis, a set of more than 150 genes were retrieved from the 5 transcriptome data, the expression of which was the most correlated with either a physical or a biochemical variable. Detailed description of these genes with their assignated function as obtained from SGD database (https://www.yeastgenome.org/) is reported in Table 2. The sPLS regression analysis further distributed this list into 6 groups based on their closest association with either one of these variables. Group 1 which contained 23 genes was positively correlated to β-1,6-glucans and to chitin but negatively associated with the contour length and β-1,3-glucans. Surprisingly enough, this cluster was enriched of genes that encode sulfate assimilation pathway which is required for the biosynthesis of cysteine, an amino acid that is implicated in the cysteine-rich domain of cell wall sensors (Kock et al., 2015) and in disulfide link between CWP. Genes that belong to the ergosterol biosynthetic process were enriched in cluster 2 which was positively correlated with hydrophobicity, adhesion events and mannans, and weakly negatively associated with the Young's modulus. On the other hand, these same biochemical and biophysical variables were negatively correlated with genes in cluster 3 from which no particular GO biological or GO component process enrichment could be found. Nonetheless, we noticed that 30% of the genes in this cluster encode proteins with unknown function. The 18 genes that constitute cluster 4 were shown to correlate positively with the Young's modulus, cell size and β-1,6-glucans content. Main biological function identified in this cluster are genes encoding proteins involved in mitotic division and chromosome separation through tubulin and microtubulin polymerization/depolymerization, which is expected as regards to the cell size parameter. Group 5 which includes 25 genes showed positive correlation with contour length and β-1,3-glucans, and this correlation could be explained by the remarkable enrichment in the seripauperin multigenes (PAU) family encoding cell wall proteins. Finally, cluster 6 that contained about 30 genes showed anti-correlation with Young modulus, cell size and to a lesser extent β-1,6-glucans. However, there was no clear GO function enrichment in this cluster that could help us to explain this negative association.

**Table 2:** Functional analysis of genes in cluster as obtained by PLS-sparse analysis of biochemical, biophysical and transcriptomic data.

**Category**	**Main biological processes identified in the cluster**		***k***	***f***
	***p*-value**	**In category from cluster**		
**CLUSTER 1**				
Sulfate assimilation [GO:0000103]	1.468e-10	MET10 MET3 MET5 MET14 MET16	5	11
Cysteine biosynthetic process [GO:0019344]	2.511e-10	MET10 MET3 MET5 MET14 MET16	5	12
Methionine biosynthetic process [GO:0009086]	5.159e-08	MET10 MET3 MET5 MET14 MET16	5	31
Methionine metabolic process [GO:0006555]	1.311e-05	MET3 MET14 MET16	3	14
Cellular amino acid biosynthetic process [GO:0008652]	1.769e-05	MET10 MET3 MET5 MET14 MET16	5	98
Transport [GO:0006810]	0.001036	OLI1 SEO1 FUR4 MET10 YGL114W AQY2 YLL053C SUL2 ZRT2	9	815
Oxidation-reduction process [GO:0055114]	0.002088	MET10 FMO1 MET5 YIM1 MET16	5	272
Transmembrane transport [GO:0055085]	0.003346	SEO1 FUR4 YGL114W SUL2 ZRT2	5	303
Sulfate assimilation, phosphoadenylyl sulfate reduction by phosphoadenylyl-sulfate reductase (thioredoxin) [GO:0019379]	0.003483	MET16	1	1
Extrachromosomal circular DNA localization involved in cell aging [GO:0034652]	0.003483	BUD6	1	1
1,6-beta-glucan metabolic process [GO:0006077]	0.003483	KRE9	1	1
Low-affinity zinc ion transport [GO:0006831]	0.003483	ZRT2	1	1
Uracil transport [GO:0015857]	0.003483	FUR4	1	1
Pyrimidine ribonucleoside biosynthetic process [GO:0046132]	0.006955	URA10	1	2
Zinc ion transmembrane transport [GO:0071577]	0.006955	ZRT2	1	2
C-terminal protein methylation [GO:0006481]	0.006955	STE14	1	2
**CLUSTER 2**				
Ergosterol biosynthetic process [GO:0006696]	7.853e-07	ERG28 ERG7 HMG1 ERG5	4	23
Steroid biosynthetic process [GO:0006694]	1.117e-06	ERG28 ERG7 HMG1 ERG5	4	25
Sterol biosynthetic process [GO:0016126]	2.079e-06	ERG28 HMG1 ERG5 CYB5	4	29
Lipid biosynthetic process [GO:0008610]	2.253e-05	ERG28 ERG7 HMG1 ERG5	4	52
Protein localization to chromosome, centromeric region [GO:0071459]	0.0001053	IML3 SCM3	2	5
Oxidation-reduction process [GO:0055114]	0.00169	HBN1 IMD2 COX8 HMG1 ERG5	5	272
Citrate transport [GO:0015746]	0.003332	PHO87	1	1
Positive regulation of translational initiation [GO:0045948]	0.003332	HYP2	1	1
Negative regulation of protein ubiquitination involved in ubiquitin-dependent protein catabolic process [GO:2000059]	0.006653	SCM3	1	2
Dephosphorylation of RNA polymerase II C-terminal domain [GO:0070940]	0.006653	RTR1	1	2
Positive regulation of translational termination [GO:0045905]	0.006653	HYP2	1	2
Coenzyme A metabolic process [GO:0015936]	0.006653	HMG1	1	2
Establishment of meiotic sister chromatid cohesion [GO:0034089]	0.006653	IML3	1	2
Positive regulation of translational elongation [GO:0045901]	0.009964	HYP2	1	3
GTP biosynthetic process [GO:0006183]	0.009964	IMD2	1	3
mRNA splice site selection [GO:0006376]	0.009964	LUC7	1	3
Isopentenyl diphosphate biosynthetic process, mevalonate pathway [GO:0019287]	0.009964	HMG1	1	3
Maintenance of meiotic sister chromatid cohesion [GO:0034090]	0.009964	IML3	1	3
**CLUSTER 3**				
Mitochondrial alanyl-tRNA aminoacylation [GO:0070143]	0.004089	ALA1	1	1
Alanyl-tRNA aminoacylation [GO:0006419]	0.008162	ALA1	1	2
Activation of adenylate cyclase activity by G-protein signaling pathway [GO:0007189]	0.008162	RAS1	1	2
Glycerol-3-phosphate catabolic process [GO:0046168]	0.008162	GPD2	1	2
**CLUSTER 4**				
Regulation of microtubule polymerization or depolymerization [GO:0031110]	0.0003118	DAD1 DUO1	2	10
Mitotic spindle organization in nucleus [GO:0030472]	0.00157	DAD1 DUO1	2	22
Protein folding [GO:0006457]	0.002074	ALF1 ZIM17 CIN2	3	96
Negative regulation of fatty acid metabolic process [GO:0045922]	0.002726	FRM2	1	1
Post-chaperonin tubulin folding pathway [GO:0007023]	0.005445	ALF1	1	2
**CLUSTER 5**				
Response to stress [GO:0006950]	2.137e-09	PAU8 PAU11 RTA1 PAU12 SSA2 PAU18 PAU23 PAU19 PAU6	9	152
Biological_process [GO:0008150]	0.0006316	COS4 PAU11 RTA1 PAU12 COS8 COS5 PAU18 PAU23 YMR122W-A PAU19 YNL155W PAU6	12	1203
Regulation of telomerase activity [GO:0051972]	0.003786	SBA1	1	1
Positive regulation of telomere maintenance via telomerase [GO:0032212]	0.007559	SBA1	1	2
**CLUSTER 6**				
G-quadruplex DNA formation [GO:0071919]	0.00424	EST1	1	1
Positive regulation of ligase activity [GO:0051351]	0.00424	ARC1	1	1
Positive regulation of ubiquitin-protein ligase activity [GO:0051443]	0.00424	DCN1	1	1
Iron assimilation by reduction and transport [GO:0033215]	0.008464	FET3	1	2
Cellular component organization [GO:0016043]	0.008464	BNR1	1	2
Formin-nucleated actin cable assembly [GO:0070649]	0.008464	BNR1	1	2
Asparaginyl-tRNA aminoacylation [GO:0006421]	0.008464	DED81	1	2
Protein targeting to peroxisome [GO:0006625]	0.008464	PEX8	1	2
tRNA aminoacylation for protein translation [GO:0006418]	0.009464	ARC1 DED81	2	35

*k refers to the number of genes in the category over the total number of genes in all clusters. f refers to the number of genes in the category over the total number of assigned genes in the genome*.

## Discussion

The purpose of this work was to unravel the connections between biochemical and biophysical properties of the cell wall, how these properties account for the molecular architecture of the wall and what are the molecular cues (i.e., genes) that are connected to these properties. We addressed this challenging question in two ways. Firstly, we investigated the cell wall composition and biophysical parameters (elasticity modulus, hydrophobicity, adhesion events with a concanavalin- functionalized AFM tips) of four industrial (diploid) strains and compared these parameters to those of the sequenced laboratory (diploid) BY4743 strain. We additionally used transcriptomic data to infer molecular explanation of the biochemical and nanomechanical properties differences between strains. This comparative analysis led us to draw simple correlations such as higher surface hydrophobicity of industrial strains associated with higher frequency of interactions between the lectin-coated AFM tips and the cell surface. This correlation could be better explained by the nature, the density and the distribution of mannoproteins at the cell surface rather than by levels of mannans. This was particularly evident for cell wall of the industrial strains L71 that has a similar amount of mannans as the laboratory strain albeit the surface hydrophobicity of these two strains was clearly different. This relation is also well documented with strain L69 that exhibits the highest hydrophobic character which coincided with the highest expression level of adhesion-encoded *FLO11* and *YHR213w* coding for a lectin-like protein similar to Flo1. Use of single-molecule AFM with tips functionalized with concanavalin A also unraveled pertinent insights on the biochemical and physical structure of the outer layer of the cell wall, as deduced from the contour length and the rupture distance. Since the contour length refers to a fully extended, but not elongated, polymer, it was expected that this value was not unique due to the high variability in mannoproteins present at the surface of the yeast. Thus, the contour lengths grossly followed a normal distribution with mean value ranging from of 20 to 100 nm. Taking into account that the mean size of a mannosyl unit is about 0.5 nm, one can estimate that the polysaccharide chains present on mannoproteins consists of 40–200 α-linked mannosyl units. Although this value is in the range of α-mannose residues in cell wall mannoproteins determined by biochemical method (Ballou, 1976; Orlean, 2012), it suggests that the size of the mannans chains that decorate cell wall proteins is different between strains, with L71 and L60 harboring the shortest (i.e., 40 mannosyl units) and strain L69, the longest (i.e., 200 units). Interestingly, this latter strain also exhibited the highest surface hydrophobicity. However, the level of protein mannosylation cannot solely account for the surface hydrophobicity since for instance the hydrophobicity of strain L71 is 5 times higher than the lab strain BY4743 albeit both strains have the same mannans content, and the size of mannans chain in mannoproteins of strain BY4743 is roughly twice larger than in strain L71. This clearly indicates that the hydrophobicity property likely depends on the type of mannoproteins at the cell surface rather than on their mannosylation levels. This suggestion is supported by the finding that strain L69 which harbors the highest hydrophobic surface characteristic has also the highest expression of *FLO11* which codes for a protein that mediates a variety of adhesive phenotypes in yeast (Dranginis et al., 2007). In addition, the product of this gene is responsible for the hydrophobic yeast “flor” that grows at the air-liquid interface (Alexandre, 2013).

Mechanical properties of the cell wall mannans at the cell surface of the yeast can also be assessed from the rupture distance which corresponds to the distance at which the binding of the polysaccharide with the AFM-tip ConA is broken. These rupture distances were in the range of 0–100 nm with elongation force fitting well with the FJC model for strain BY4743 and industrial strains L71, L60, and L69. This may suggest that the mannans chains are mechanically stretched from the cell surface when pulled away by the AFM-tip ConA. In contrast, elongation forces on the cell surface of L69 strain were better described by a WLC model, and resulted in rupture distances as large as 400 nm, which led to propose that the entire mannoproteins and not solely α-mannan chains were mechanically stretched out from the cell wall. In conclusion, this single-molecule AFM study highlighted two relevant data, namely that the α-mannan oligosaccharides that decorate cell wall proteins and that the anchorage of these mannoproteins in the wall can be very different between yeast strains, which may reflect their environmental adaptation.

Whilst this comparative analysis of the biochemical composition and elasticity of cell wall pointed out clear differences between strains that could be in part explained by variance in gene expression, this approach does not permit to explore connections between these properties and how these properties can be molecularly explained. Use of multivariate methods such as Sparse partial least squares (sPLS) or sPLS discriminant analysis (DA) are well dedicated to extract correlations between apparently heterogeneous datasets in which the number of variables (e.g., genes, proteins, metabolites, etc.) is larger than samples (e.g., strains, test conditions, etc.). Applying these biostatistical methods (Le Cao et al., 2009b, 2011), we found a close connection between β-1,3-glucans and contour length, indicating that the physical extension of α-mannan chains on mannoproteins depends on the linkage of these proteins to β-1,3-glucans, in accordance with the fact that a majority of cell wall proteins are linked directly or indirectly to the β-1,3-glucan network (Klis et al., 2006). Interestingly, proteins encoded by seripauperin (*PAU*) genes may contribute to this physical property of the cell wall since several of them are found to positively correlate with the contour length. On the contrary, the negative correlation of contour length with a set of genes whose main biological process was related to sulfur metabolism would suggest that the mechanical properties of the cell wall is negatively impacted by proteins that are weakly retained via disulfide bridges. As expected, we found a strong association between hydrophobicity and adhesion events. These two physical properties and the biochemical variable mannans were found to positively correlate with a set of genes whose main GO function was related to ergosterol metabolism. This finding is suggestive of a linkage between physical properties of cell wall and membrane structure and/or fluidity. Furthermore, the negative correlation of Young's modulus with this set of genes comprising ergosterol encoded genes supports our recent work showing that plasma membrane contributes to the cell wall stiffness properties (Schiavone et al., 2016). This correlation also agreed with the finding that uptake of sterol is favored by highly mannosylated proteins encoded by TIR/DAN genes family (Abramova et al., 2001; Francois, 2016).

## Conclusion

In this work, a combined biochemical, biophysics and molecular analysis has been undertaken on 5 different yeast strains (4 industrial and the laboratory sequenced genome BY4743 strains) with the eventual purpose to identify molecular cues that determine the structural and nanomechanical properties of the yeast cell wall. While this study reinforces our previous suggestions (Dague et al., 2010; Schiavone et al., 2015) that the linkages between cell wall polysaccharides components contribute to the physical (contour length, elasticity) properties, our multivariate analysis demonstrated that the elasticity property of the yeast wall is merely dependent on molecular connections between mannoproteins and the β-1,3-glucan network. This integrated analysis also highlights that the surface hydrophobicity is determined by the abundance of a certain type of mannoproteins, and notably adhesin or adhesin-like encoded, respectively, by *FLO11* and *YHR 313w* genes and not by the mannan content *per se*. Finally, this integrated analysis confirms a role of the membrane structure/fluidity in elasticity of the cell wall and it underscores a potential implication of the sterol metabolism in the surface hydrophobicity property of the cell wall. We anticipate that investigation of other yeast strains and other cultures/process conditions shall enrich this multivariate statistical analysis, and hence, unravel in a deeper way the connection between genetic markers and cell wall biochemical and physical properties. In an applied view point, identifying genetic markers that can impact cell wall composition and properties would be exploited for strains engineering and improvement with respect to their dedicated final utilization.

## Author contributions

MS and JF designed the experiments. MS carried out the biological and AFM experiments. SD contributed to the statistical analysis, ED to the AFM experiments. NS and MC contributed to the interpretation of the analysis with respect to industrial context. MS and JF wrote the manuscript which was improved and approved by all other authors.

### Conflict of interest statement

The authors declare that the research was conducted in the absence of any commercial or financial relationships that could be construed as a potential conflict of interest.

## Supplementary material

Figure S1 Extension of the linear polymer by the worm-like chain (WLC) and freely-jointed chain (FJC) models. Force vs. distance curves predicted by the model WLC for L69 **(A)** and for the FJC model for L71 strain **(B)**. Distribution of the persistent length **(C)** and Kuhn length values **(D)**. Representation of the unbinding forces measured from all force-distance curves vs. persistent length values **(E)** or Kuhn length values **(F)**.

Figure S2 Representation of the samples after a Sparse PLS regression analysis of transcriptome data obtained from the laboratory and industrial strains. For each strain, the transcriptome data were obtained from three independent biological replicate and the normalized data were used in Sparse PLS regression.

Figure S3 Venn representation of differentially expressed genes in the industrial strains compared to reference strains. The number of differentially genes between each strain is reported in the ellipses.

Figure S4 Functional analysis of differentially expressed genes in industrial strains as compared to the laboratory strain BY4743. Functional analysis was carried out using Funspec (http://funspec.med.utoronto.ca/) using *p*-value of 0.01 with Bonferroni correction with the 71 common genes that were differentially expressed in the 4 industrial strains as compared to the laboratory strain.

Figure S5 Detailed GO term analysis of differentially expressed genes related to cell wall function in industrial strains. Functional analysis of differentially expressed genes related to cell wall (81) in Figure 6 was carried out using Funspec at (http://funspec.med.utoronto.ca/) using *p*-value < 0.01 with Bonferroni correction.

Figure S6 Correlation circle plot with biochemical-biophysical analysis of industrial yeasts. The plots represent in component 1 the genes whose absolute expression level is the most correlated with the physico-chemical and biochemical variables represented as component 2.
