# Supplementary material for: Integration of Biochemical, Biophysical and Transcriptomics Data for Investigating the Structural and Nanomechanical Properties of the Yeast Cell Wall
Source: Front Microbiol. 2017 Sep 27;8:1806. doi: 10.3389/fmicb.2017.01806 (PMC5649194; doi:10.3389/fmicb.2017.01806)

Figure S1 (Schiavone et al.)

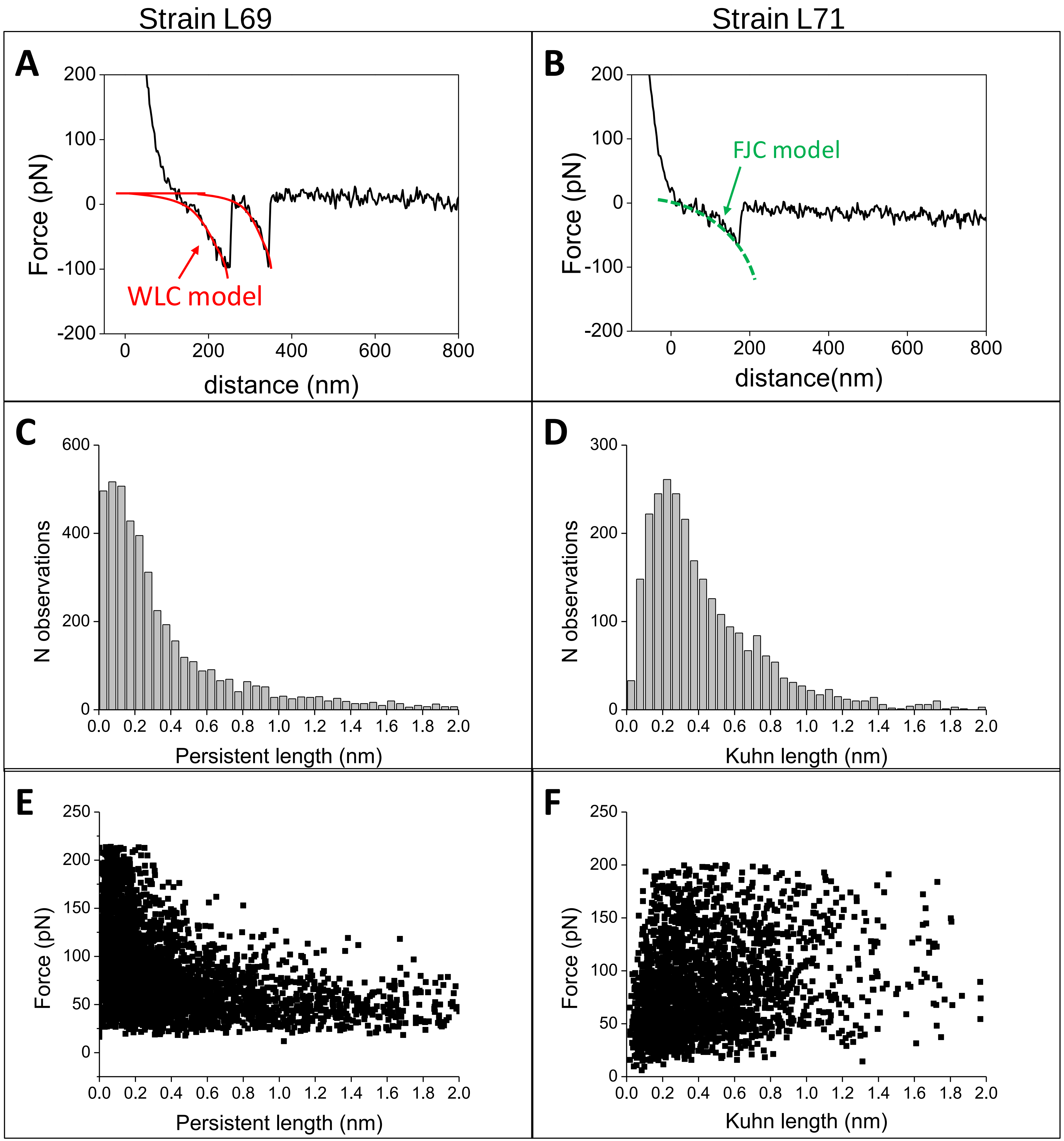

Figure S2 (Schiavone et al.)

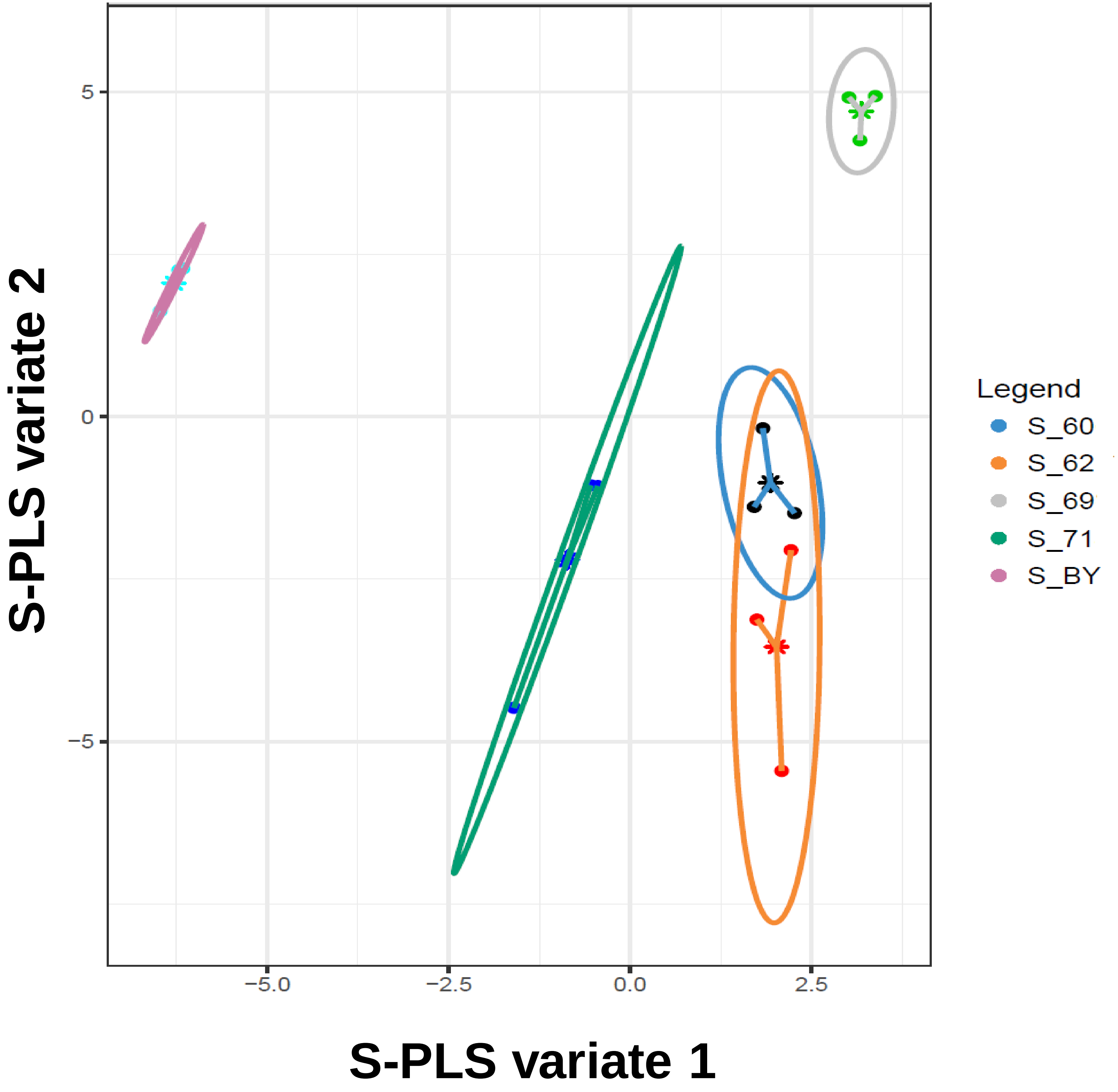

Figure S3 (Schiavone et al.)

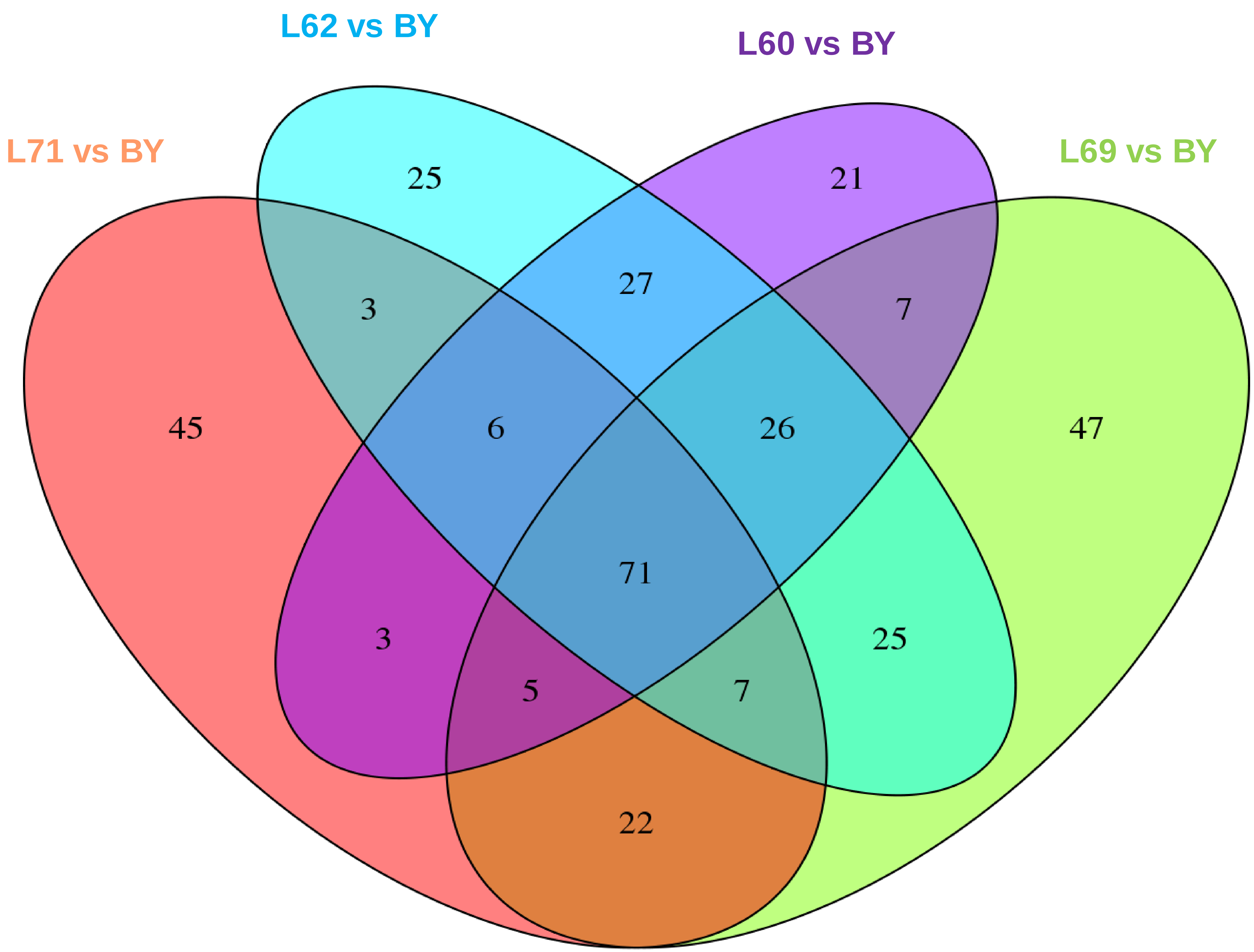

Figure S4 (Schiavone et al.)

A. Differentially expressed genes in industrial strains vs lab strain according to G0-molecular function

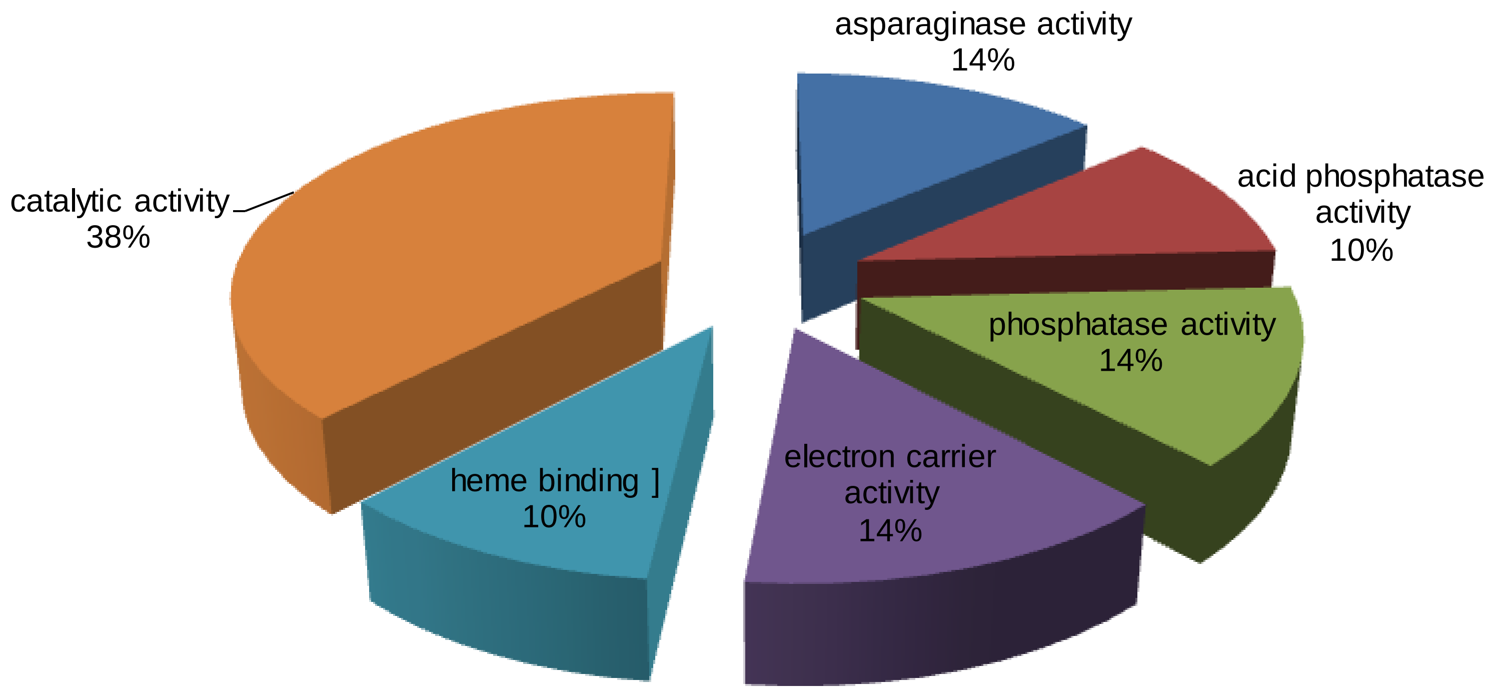

B. Differentially expressed genes in industrial strains versus laboratory strain according to GO cellular component

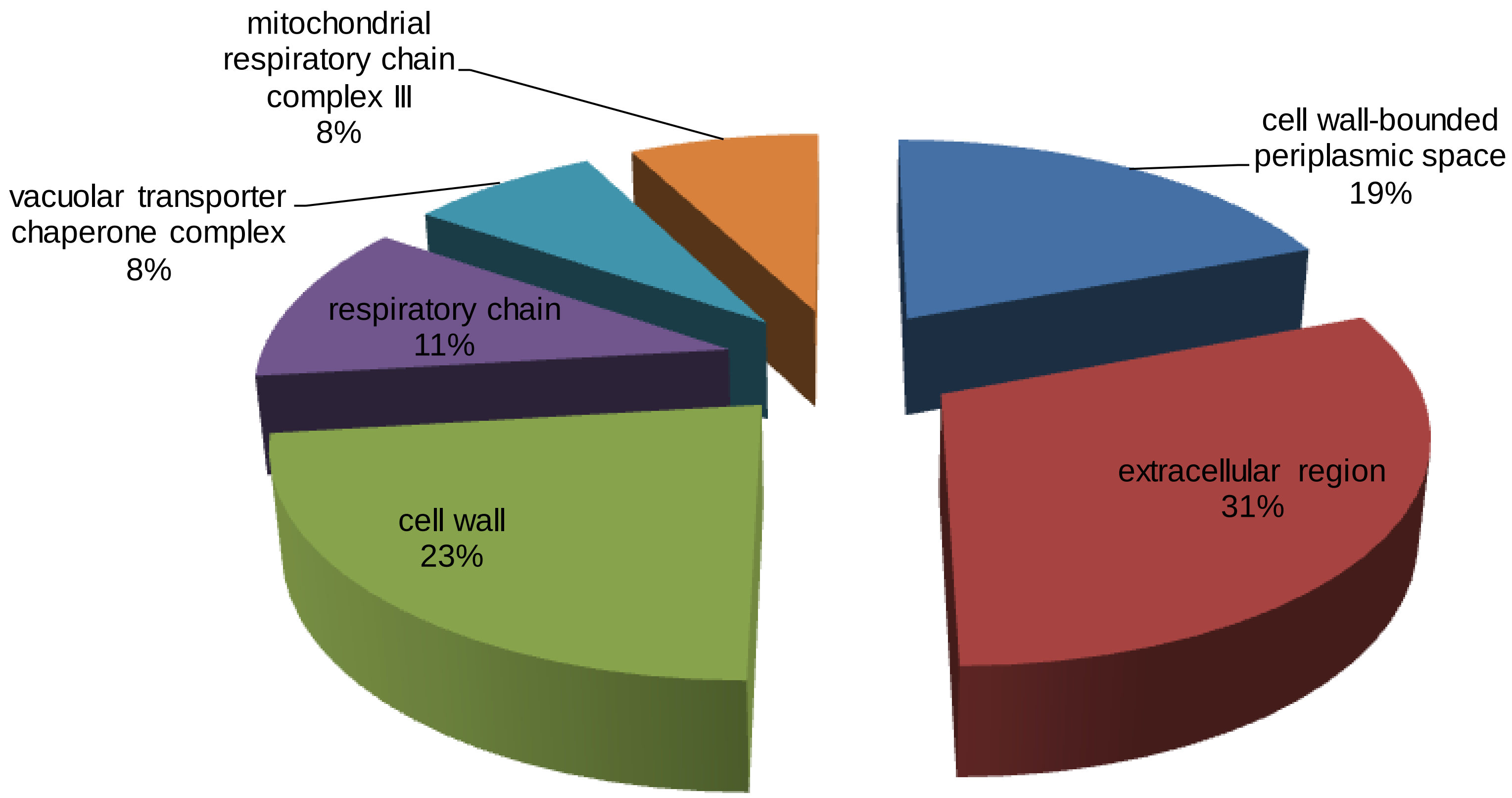

Figure S5 (Schiavone et al.)

A. Differentially expressed genes in industrial strains versus lab strain according to GO molecular function

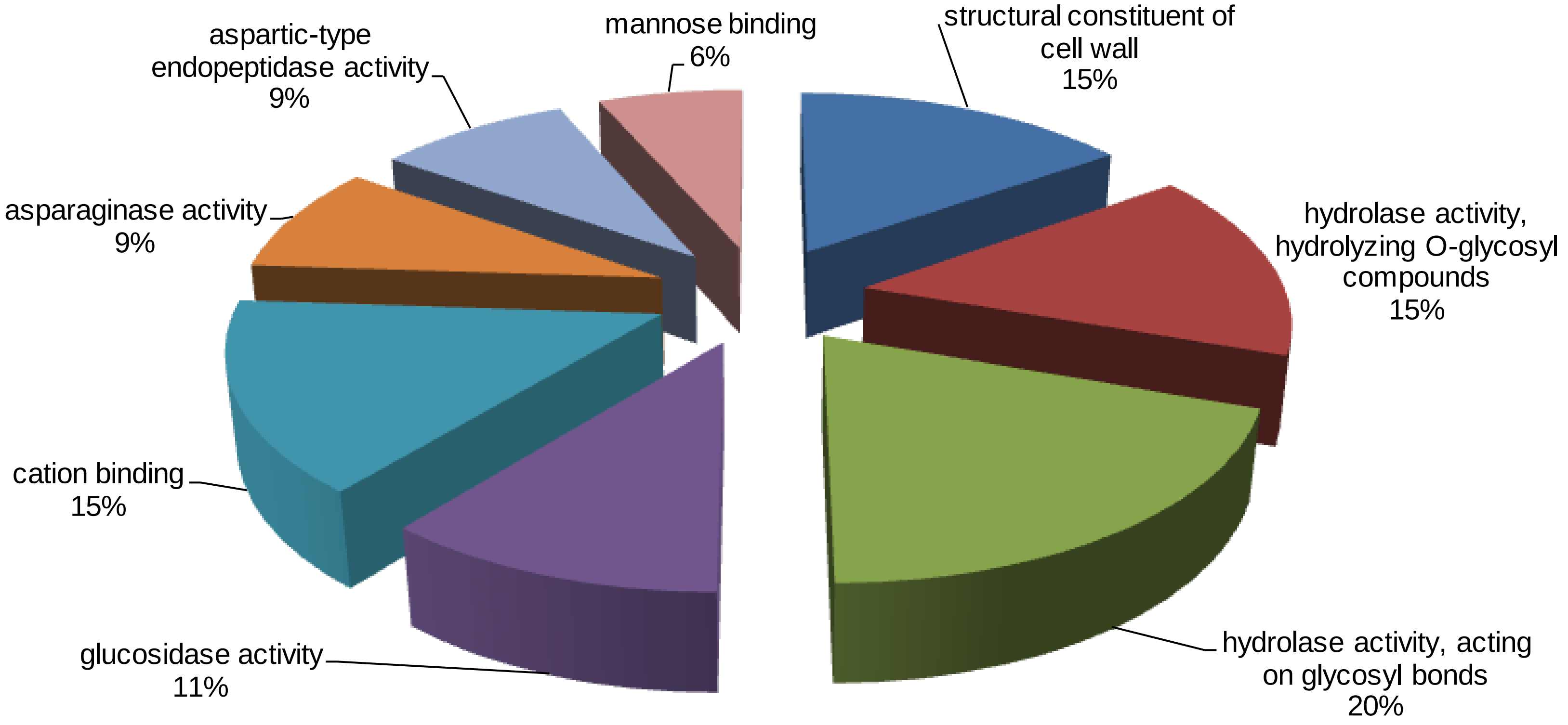

B. Differentially expressed genes in industrial strains versus lab strain according to GO biological term

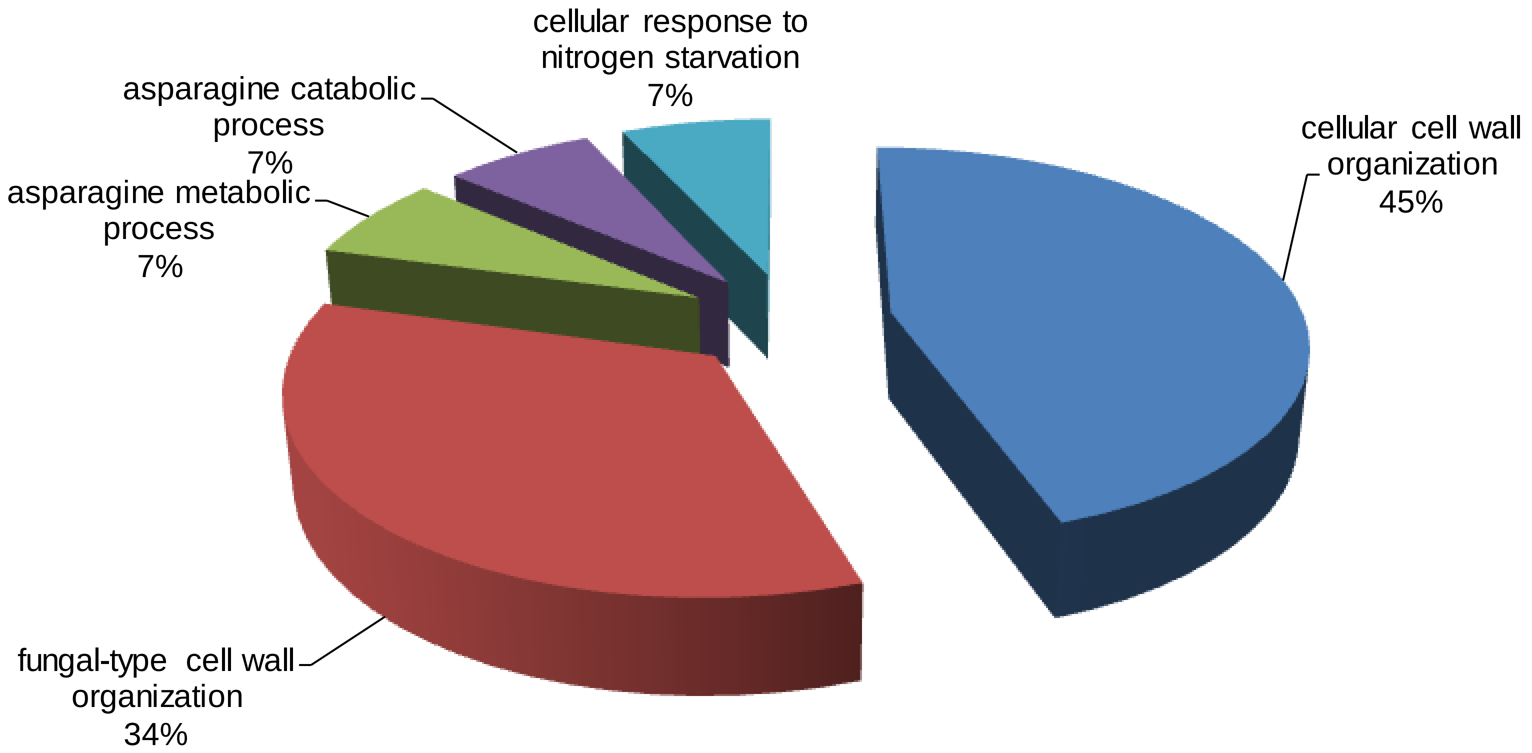

Figure S6 (Schiavone et al.)

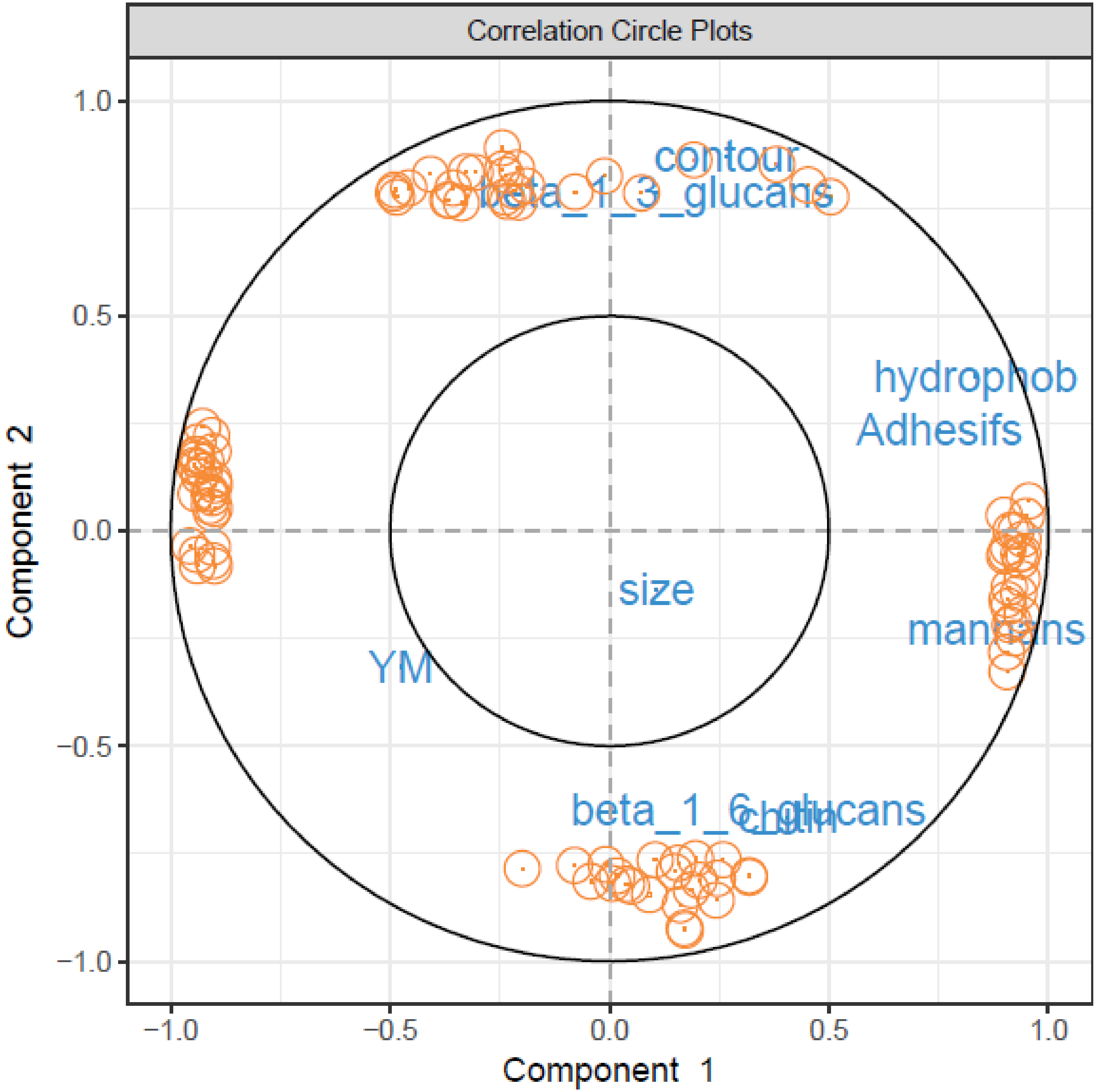

Supplement: Figure S1 — Extension of the linear polymer by the worm-like chain (WLC) and freely-jointed chain (FJC) models. Force vs. distance curves predicted by the model WLC for L69 (A) and for the FJC model for L71 strain (B). Distribution of the persistent length (C) and Kuhn length values (D). Representation of the unbinding forces measured from all force-distance curves vs. persistent length values (E) or Kuhn length values (F). [file Presentation1.PDF]
